# CLUH maintains functional mitochondria and translation in motoneuronal axons and prevents peripheral neuropathy

**DOI:** 10.1126/sciadv.adn2050

**Published:** 2024-05-29

**Authors:** Marta Zaninello, Tim Schlegel, Hendrik Nolte, Mujeeb Pirzada, Elisa Savino, Esther Barth, Ines Klein, Hauke Wüstenberg, Tesmin Uddin, Lisa Wolff, Brunhilde Wirth, Helmar C. Lehmann, Jean-Michel Cioni, Thomas Langer, Elena I. Rugarli

**Affiliations:** ^1^ Institute for Genetics, University of Cologne, Cologne 50931, Germany.; ^2^ Cologne Excellence Cluster on Cellular Stress Responses in Aging-Associated Diseases (CECAD), Cologne 50931, Germany.; ^3^ Max Planck Institute for Biology of Ageing, Cologne 50931, Germany.; ^4^ Division of Neuroscience, IRCCS San Raffaele Scientific Institute, Milan 20132, Italy.; ^5^ Department of Neurology, University of Cologne, Cologne 50931, Germany.; ^6^ Institute of Human Genetics, University of Cologne, Cologne 50931, Germany.; ^7^ Center for Molecular Medicine (CMMC), University of Cologne, Cologne 50931, Germany.; ^8^ Center for Rare Diseases Cologne (CESEK), University Hospital of Cologne, Cologne 50937, Germany.

## Abstract

Transporting and translating mRNAs in axons is crucial for neuronal viability. Local synthesis of nuclear-encoded mitochondrial proteins protects long-lived axonal mitochondria from damage; however, the regulatory factors involved are largely unknown. We show that CLUH, which binds mRNAs encoding mitochondrial proteins, prevents peripheral neuropathy and motor deficits in the mouse. CLUH is enriched in the growth cone of developing spinal motoneurons and is required for their growth. The lack of CLUH affects the abundance of target mRNAs and the corresponding mitochondrial proteins more prominently in axons, leading to ATP deficits in the growth cone. CLUH interacts with ribosomal subunits, translation initiation, and ribosome recycling components and preserves axonal translation. Overexpression of the ribosome recycling factor ABCE1 rescues the mRNA and translation defects, as well as the growth cone size, in CLUH-deficient motoneurons. Thus, we demonstrate a role for CLUH in mitochondrial quality control and translational regulation in axons, which is essential for their development and long-term integrity and function.

## Teaser

CLUH regulates mitochondrial function in peripheral motor axons, thus promoting growth and preventing degeneration.

## INTRODUCTION

Neurons are highly polarized cells with distinct dendritic arborizations and axonal projections that can extend for long distances. Efficient trafficking of mitochondria and robust quality control mechanisms are essential to maintain the supply and the functionality of these organelles at distal sites, such as the growing tips of developing axons and the synaptic terminals in the mature nervous system (*1*). The long journey of mitochondria to reach peripheral positions makes them susceptible to depletion of short-lived proteins, less flexible to meet metabolic demands via proteome adaptation, and at risk of unbalanced stoichiometry of nuclear- and mitochondrially encoded subunits of the respiratory chain (*1*). Local translation of mitochondrial proteins in axons is a safeguard mechanism to counteract mitochondrial ageing and preserve their function (*2*–*4*). mRNAs for mitochondrial proteins can be detected in axons, where they are associated with organelles, such as mitochondria and endosomes (*5*–*8*), or in RNA granules in complex with RNA binding proteins (RBPs) (*9*). Several mRNAs for mitochondrial proteins were found to belong to a category of highly translated mRNAs in axons in the adult mammalian nervous system (*10*). However, knowledge of the ribonucleoprotein code that regulates the axonal transport, stability, and translation of these mRNAs is still sparse. Elucidating the involved molecular components is key to understand how neurons maintain a functional pool of mitochondria throughout a lifetime.

Clustered mitochondria homolog (CLUH) is an evolutionarily conserved cytosolic RBP, which binds to a wide range of mRNAs encoding mitochondrial proteins involved in oxidative phosphorylation (OXPHOS), the tricarboxylic acid (TCA) cycle, and multiple metabolic pathways (*11*, *12*). In several organisms, CLUH loss induces mitochondrial clustering, reshapes the mitochondrial proteome, and leads to respiratory defects (*11*, *13*–*18*). Constitutive lack of CLUH in human cells and mouse liver is associated with a down-regulation of its target mRNAs, which are subjected to enhanced decay (*16*, *19*). In vivo, CLUH function is crucial at metabolic switches, such as the fetal-neonatal transition, starvation, or adipogenesis (*16*, *20*). Although several lines of evidence suggest a role for CLUH in translational regulation (*11*, *18*, *20*, *21*), how this occurs is an open question. Here, we examined the role of CLUH in neurons whose integrity depends on RNA transport and local translation. Ablation of CLUH expression in mouse neural progenitors revealed a role in maintaining motor behavior, by preventing late-onset degeneration of distal axons and neuromuscular junctions (NMJs). In vitro, CLUH is essential for axonal growth, maintains functional mitochondria at the growth cone (GC), and sustains axonal translation. CLUH associates with components involved in initiation of translation and ribosome recycling and is essential to preserve these components in axons. We find that overexpression of ATP Binding Cassette Subfamily E Member 1 (ABCE1), which is involved in ribosomal quality control and translational initiation, rescues the *Atp5a1* mRNA and translational defect observed in CLUH-deficient motoneuronal axons, as well as the GC size. Our data link CLUH to translational quality control and demonstrate the importance of this process in maintaining functional mitochondria in distal axons.

## RESULTS

### *Cluh* deletion in the mouse neural progenitors impairs locomotor activity and causes peripheral neuropathy

**Fig. 1.**
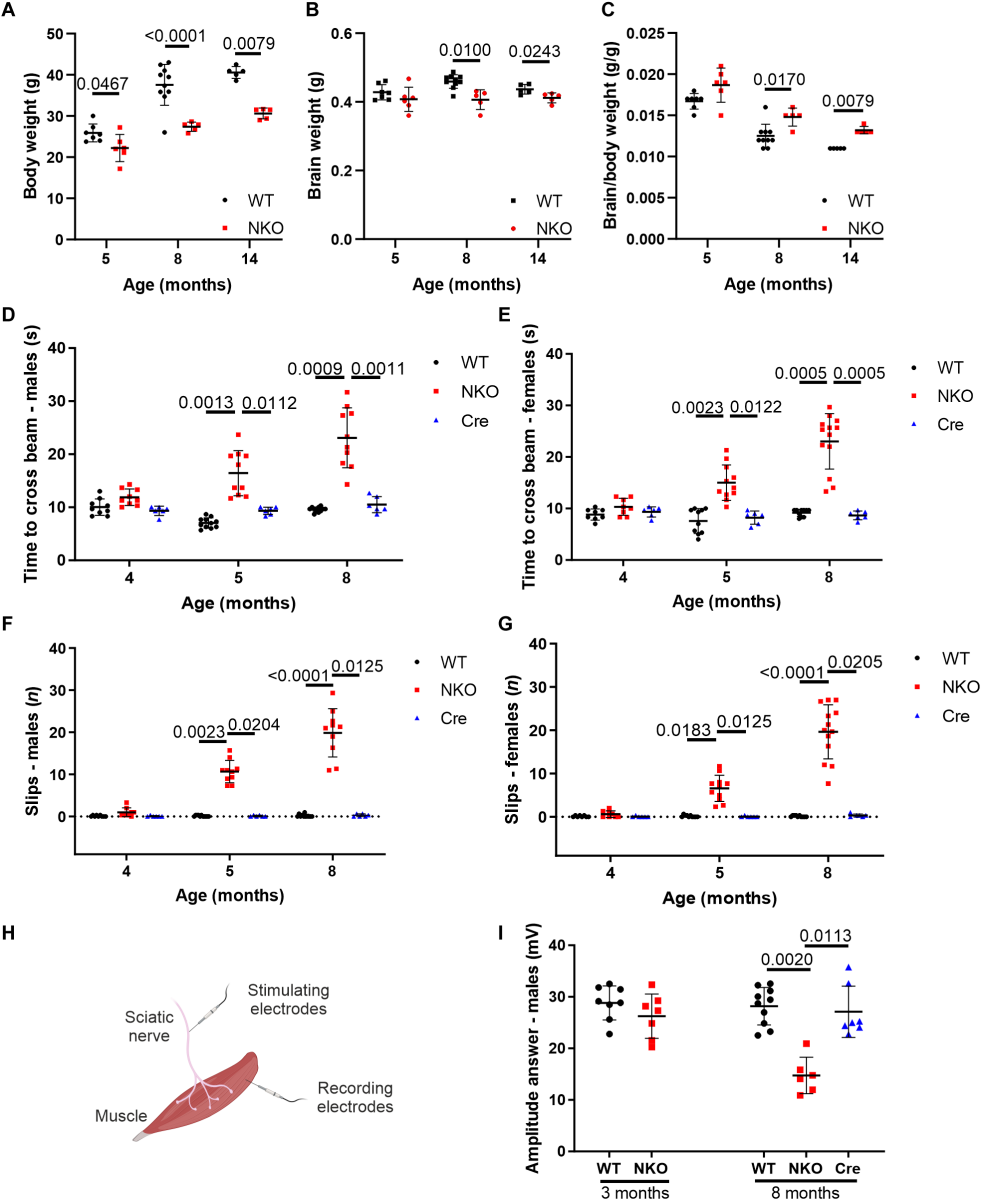
NKO mice show progressive locomotor defects. (**A** to **C**) Body weight (A), brain weight (B), and brain/body weight ratio (C) of WT and NKO mice at 5, 8, and 14 months of age. Data represent means ± SD of 5 to 10 mice. Statistical significance was determined by Mann-Whitney and Welch’s *t* tests. (**D** to **G**) Quantification of the walking beam test in males [(D) and (F)] and females [(E) and (G)] aged 4, 5, and 8 months as time spent to cross the beam [(D) and (E)] and number of total slips during the crossing [(F) and (G)]. Data represent means ± SD of 6 to 13 mice. Statistical significance was determined by Mann-Whitney and Welch’s *t* tests. (**H**) Scheme depicting compound muscle action potential (CMAP) recording. Sciatic nerve was stimulated with two electrodes, and the action potential was measured with two recording electrodes in the muscle of the hind paw. Figure created with Biorender.com. (**I**) Quantification of the CMAP amplitude recorded in the hind paw after stimulation of the sciatic nerve of male mice aged 3 and 8 months. Data represent means ± SD of 6 to 10 mice. Statistical significance was determined by Welch’s *t* test and one-way analysis of variance (ANOVA) followed by Dunn’s multiple comparisons test.

Cortical and spinal motoneurons, which control voluntary movements, are endowed with the longest axons in the central nervous system and are prone to degenerate when local translation or mitochondrial function is impaired (*1*, *21*, *22*). To investigate whether CLUH is required for the maintenance of neuronal circuits in vivo, we knocked out the gene in mouse neural progenitors, by crossing previously characterized mice carrying *Cluh* alleles with a loxP-flanked exon 10 (*16*) with nestin-Cre transgenic mice (*23*) (genotype: *Cluh*^fl/fl^ nestin-Cre^tg/+^ from now on indicated as NKO). CLUH expression was successfully ablated in the forebrain and cerebellum and substantially reduced in the spinal cord (fig. S1A). NKO mice were viable and fertile but had a smaller body weight and a slightly increased brain weight when normalized by the body weight (Fig. 1, A to C). This result is in agreement with the known hypopituitarism of the nestin-Cre line (*24*). Motor behavior was normal at 4 months, but, at 5 months, NKO mice showed locomotor impairment, as revealed by a classical walking beam test, a proxy of the motor coordination of hindlimbs (Fig. 1, D to G, and fig. S1, B and C). NKO mice were slower and slipped more frequently while crossing the beam, independently of their gender (Fig. 1, D to G, and movies S1 to S3). The effect of *Cluh* deletion was more severe 3 months later (Fig. 1, D to G, and movies S1 to S3), indicating a progressive phenotype. The motor deficit was confirmed by a reduced latency time of NKO mice to fall in the accelerating rotarod test, which measures grip, motor coordination, and balance, with males showing a more severe phenotype than females in this test (fig. S1, B and C). In all experiments, nestin-Cre mice (Cre) were also tested to exclude any effect due to the expression of the Cre recombinase (*25*).

**Fig. 2.**
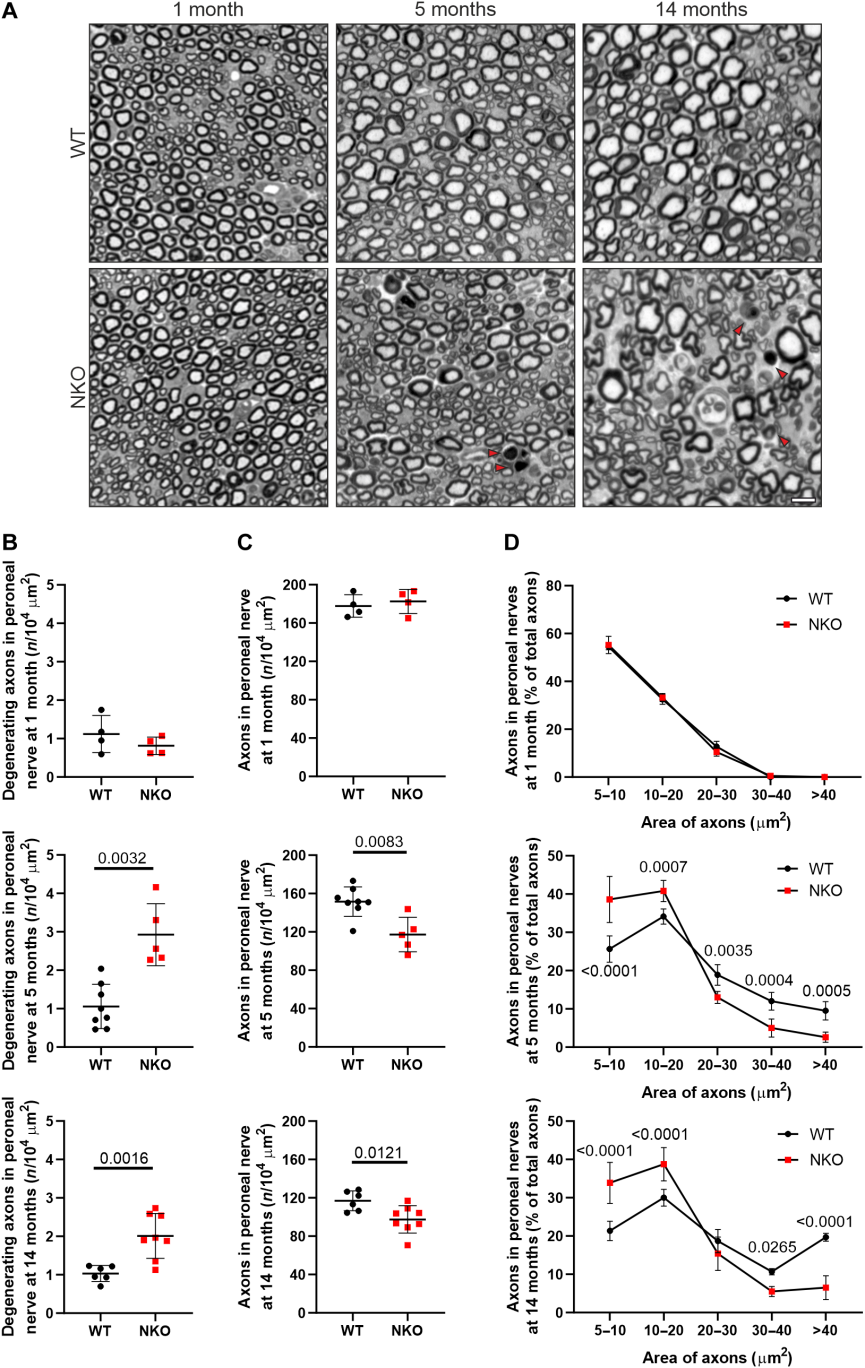
NKO mice show peripheral neuropathy. (**A**) Semithin sections of the peroneal branch of the sciatic nerve of 1-, 5-, and 14-month-old mice. Arrowheads indicate degenerating axons. Scale bar, 10 μm. (**B** to **D**) Quantification of degenerating axons (B), of the number of axons larger than 5 μm^2^ per area (C), and of the distribution of axons of different size (D) in semithin sections of the peroneal branch of the sciatic nerve at indicated ages and genotypes. Data represent means ± SD of five to eight mice. Statistical significance was determined by Welch’s *t* test in (B) and (C) and by two-way ANOVA followed by Sidak’s multiple comparisons test in (D).

**Fig. 3.**
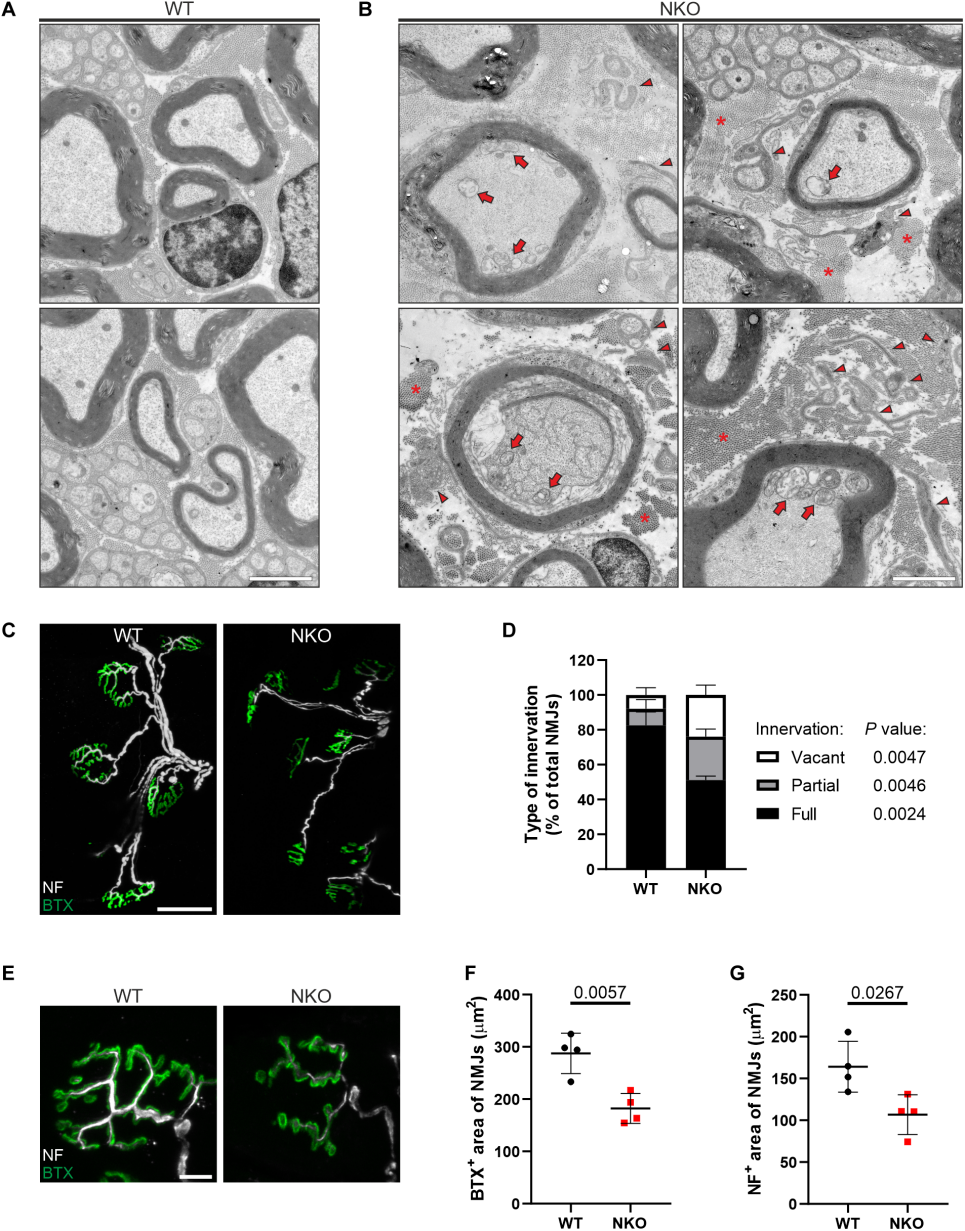
NKO mice have ultrastructurally abnormal axons and denervated NMJs. (**A** and **B**) Electron micrographs of peroneal nerves of WT (A) and NKO (B) mice aged 14 months. Arrows indicate abnormal swollen mitochondria, asterisks show accumulation of collagen, and arrowheads point to denervated Schwann cells. Scale bars, 2 μm. (**C**) NMJs of tibialis anterior muscles in WT and NKO mice aged 14 months. NF, neurofilament; BTX, α-bungarotoxin. Scale bar, 50 μm. (**D**) Quantification of the morphology of NMJs in experiments as in (C). Data represent the means ± SD of four mice (70 to 107 NMJs per mouse). Statistical significance was determined by Welch’s *t* test. *P* values respect to type of innervation are indicated. (**E**) Enlarged images of NMJs of tibialis anterior muscles in WT and NKO mice aged 14 months. Scale bar, 10 μm. (**F** and **G**) Quantification of the BTX^+^ area of post synapses (F) and NF^+^ area of pre-synapses (G) in experiments as in (C). Data represent the means ± SD of four mice (53 to 91 NMJs per mouse). Statistical significance was determined by Welch’s *t* test.

The motor impairment of NKO mice suggested a peripheral neuropathy. To test this hypothesis, we performed electrophysiological studies to record the sum of action potentials [compound muscle action potential (CMAP)] generated in the muscles of the hind paw, after stimulating the sciatic nerve that contains the axons of spinal motoneurons (Fig. 1H). In NKO muscles, the CMAP amplitude, which is a reliable index of axonal damage, was comparable to wild type (WT) at 3 months but was halved at 8 months (Fig. 1I). To substantiate these findings, we collected the distal part of the sciatic nerve, where it separates in the tibial and peroneal branches and performed semithin sections at earlier and later ages. At 1 month of age, while the NKO nerves contained a similar number of axons of comparable size (Fig. 2, A to D), at 5 and 14 months, we observed a pathological phenotype especially pronounced in the peroneal branch of the NKO nerve (Fig. 2, A to D). This was characterized by a decreased number of axons per area, with an increase of smaller axons at the expense of large axons and the appearance of profiles suggesting axonal degeneration (Fig. 2, A to D). Ultrastructurally, the NKO nerves were characterized by basal lamina–covered Schwann cell profiles devoid of axons and myelin that most probably represent denervated Schwann cells (reminiscent of bands of Büngner), increased connective tissue with collagen deposition, and collagen pockets, which are more often observed with loss of small unmyelinated fibers (Fig. 3, A and B). Moreover, we found myelinated fibers with accumulated organelles or containing swollen mitochondria, indicating impaired axonal trafficking preceding degeneration (Fig. 3B). Mitochondria in NKO axons showed more frequently open cristae than in WT (fig. S2A). Axonal pathology was supported by transcriptome changes detected in the sciatic nerve at 5 months of age. Transcripts involved in neurotransmitter regulation and synaptic function were decreased in NKO nerves, whereas mRNAs coding for pro-survival and inflammatory components were up-regulated, in agreement with a degenerative program (table S1 and fig. S2B).

To further characterize the phenotype of motor axons, we analyzed the NMJs in the tibialis anterior muscle, which is innervated by the peroneal nerve. NMJs are specialized synapses of spinal motoneurons with the skeletal muscle, whose size affects motor performance and diminishes with aging and motor neuron diseases (*26*). We detected an increased number of denervated or poorly innervated NMJs at 14 months of age (Fig. 3, C and D). The innervation and postsynaptic areas of NKO NMJs were smaller than in WT mice (Fig. 3, E to G). In contrast, the number of spinal motoneurons positive for choline acetyltransferase (ChAT) was unaffected in NKO spinal cords (fig. S2, C and D), indicating that loss of CLUH induces specifically an axonopathy. Together, our data identify a crucial role of CLUH in vivo to support motor behavior, preserve motor axon function in the sciatic nerve, and prevent loss of axons.

### CLUH regulates the size and the mitochondrial ATP levels of GCs of spinal motoneurons

**Fig. 4.**
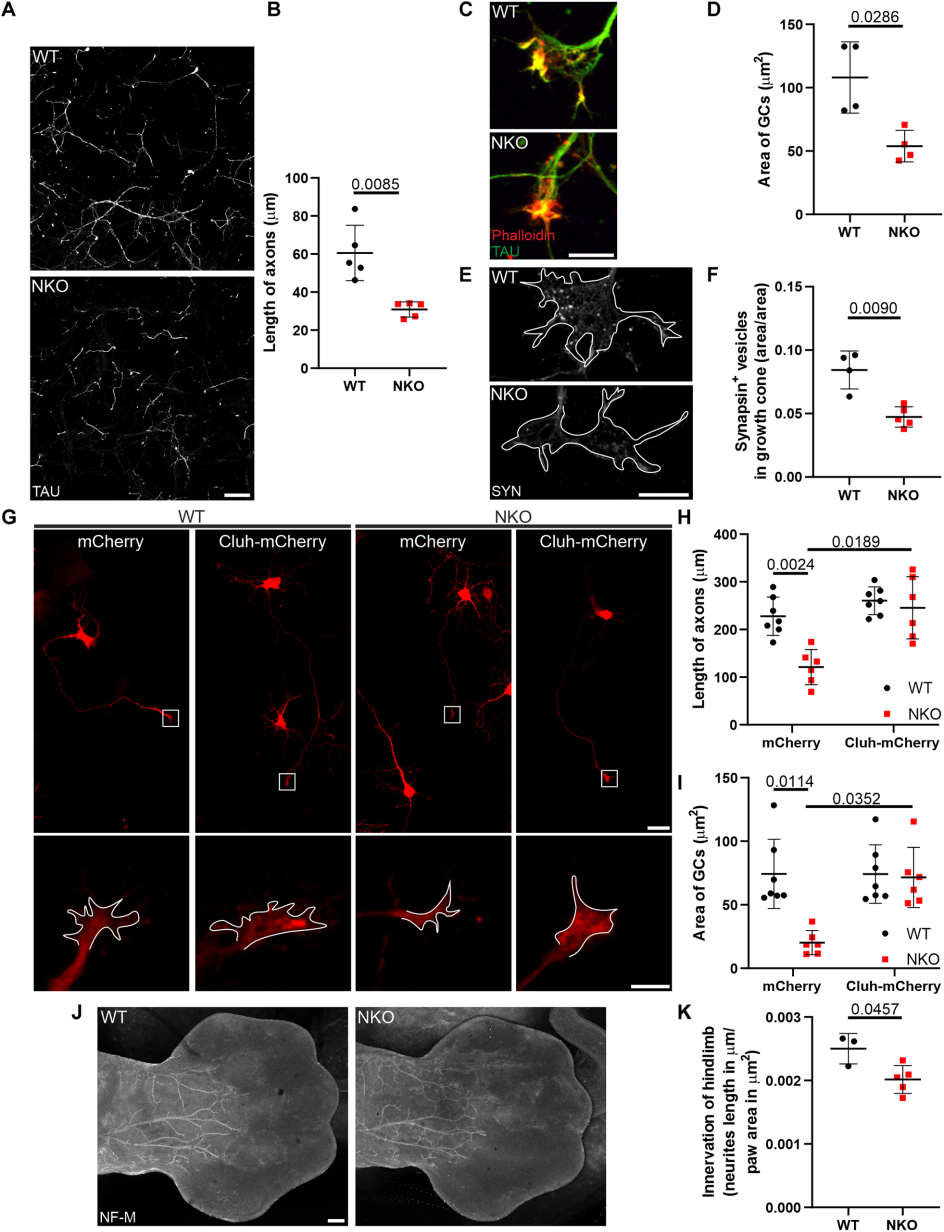
Lack of CLUH impairs axonal growth. (**A**) Motoneuronal axons stained by TAU in the axonal compartment of Boyden chambers at DIV 6. Scale bar, 75 μm. (**B**) Quantification of the length of axons in experiments as in (A). Data represent the means ± SD of five mice (four to five fields per mouse). Statistical significance was determined by Welch’s *t* test. (**C**) GCs of primary spinal motoneurons stained for TAU (green) and actin (Phalloidin, red). Scale bar, 10 μm. (**D**) Quantification of the area of GCs in experiments as in (C). Data represent the means ± SD of four mice (47 to 78 GCs per mouse). Statistical significance was determined by Mann-Whitney test. (**E**) Staining of synapsin^+^ (SYN) vesicles in GCs of primary spinal motoneurons. GCs are outlined in white. Scale bar, 10 μm. (**F**) Quantification of the number of SYN^+^ vesicles in GCs in experiments as in (E). Data represent the means ± SD of four to five mice (26 to 35 GCs per mouse). Statistical significance was determined by Welch’s *t* test. (**G**) WT and NKO primary motoneurons expressing mCherry or Cluh-mCherry. GCs are magnified in the bottom panels. Scale bars, 20 and 5 μm. (**H** and **I**) Quantification of the length of axons (H) and size of GCs (I) in experiments as in (G). Data represent the means ± SD of six to seven mice (6 to 14 axons or GCs per mouse). Statistical significance was determined by one-way ANOVA followed by Dunn’s and Dunnett’s multiple comparisons tests. (**J**) Whole mounts of hindlimbs of E13.5 WT and NKO embryos stained with neurofilament M (NF-M). Scale bar, 200 μm. (**K**) Quantification of the innervation of the hindlimbs in respect to the area of the paw as in (J). Embryos belonged to the same litter. Data represent the means ± SD of three to five mice. Statistical significance was determined by Welch’s *t* test.

To understand whether the axonal phenotype triggered by loss of CLUH in vivo is cell-autonomous, we turned to in vitro experiments using primary embryonic spinal motoneurons cultured in the absence of glia. After 6 days in vitro (DIV), NKO motoneurons displayed shorter axons compared to WT motoneurons (Fig. 4, A and B) and, at DIV 10, started to die, as assessed by a decreased number of TAU^+^ (tubulin-associated unit) and increased number of terminal deoxynucleotidyl transferase–mediated deoxyuridine triphosphate nick end labeling (TUNEL)^+^ neurons (fig. S3, A to D). For this reason, we performed all subsequent experiments in vitro at DIV 6. CLUH levels did not change during neuronal maturation (fig. S3E), and the expression of the motoneuronal markers *Hb9* and *ChAT* (*27*) was not affected in the absence of CLUH (fig. S3F). Neither the axonal density of mitochondria nor the neuronal mt-DNA levels were affected by loss of CLUH (fig. S4, A to C). Moreover, WT and NKO mitochondria in axons were comparable for morphology and membrane potential (fig. S4, D and E) (*28*). However, the size of GCs and their content of synaptic vesicles was reduced in NKO motoneurons (Fig. 4, C to F), consistent with a growth defect. To control for the specificity of this phenotype, we transfected primary motoneurons with a vector expressing the mouse isoform of CLUH tagged with a mCherry fluorescent moiety at the C terminus (fig. S5A). Overexpressed CLUH was highly enriched in the soma but was also present along the axons, especially close to axonal protrusions, and was enriched in the peripheral part of GCs (fig. S5, B and C), which contains tyrosinated tubulin, IQ motif containing GTPase activating protein 1 (IQGAP1) and cytoplasmic linker associated protein 2 (CLASP2), but not acetylated tubulin (fig. S5D) (*29*). We confirmed that an untagged human CLUH construct was similarly located at the GC (fig. S5E) and that an empty mCherry vector did not produce the same GC staining (fig. S5F). Overexpression of CLUH rescued both axonal length and GC size of NKO motoneurons (Fig. 4, G to I).

The axonal grow phenotype observed in vitro prompted us to evaluate whether axonal growth defects occurred also in vivo as a delay in innervation, which may be later compensated. Notably, developing axons in the hindlimbs labeled by neurofilament antibodies in whole-mount embryonic day 13.5 (E13.5) NKO embryos were shorter than in WT (Fig. 4, J and K).

**Fig. 5.**
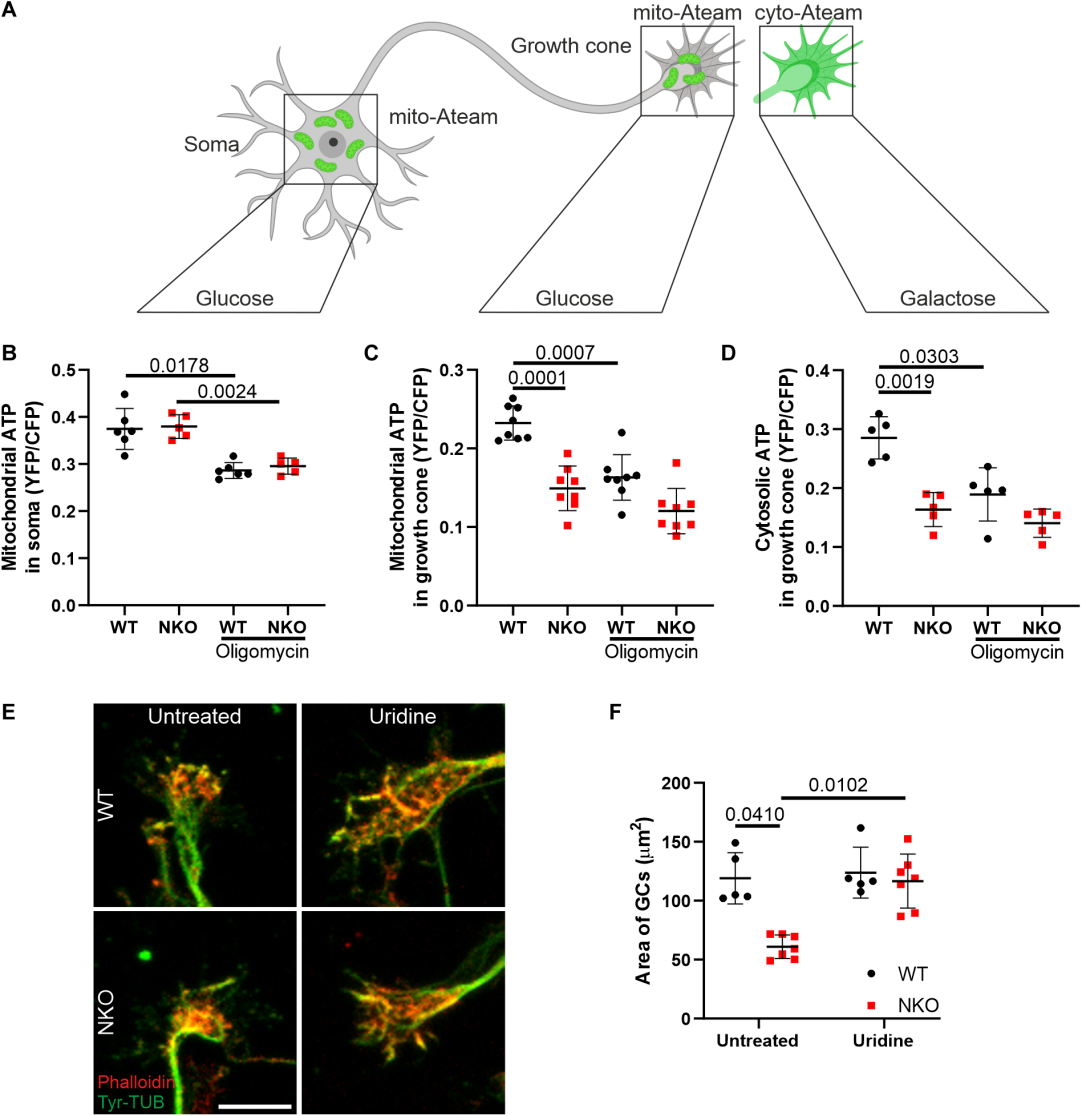
Lack of CLUH leads to ATP defects in GCs. (**A**) Depiction of the experimental strategy using the ATP sensor mito-ATeam targeted to mitochondria or the cytosol. Figure created with Biorender.com. (**B** and **C**) Quantification of mitochondrial ATP in somas (B) and GCs (C) of primary motoneurons transfected with mito-ATeam. The analyzed somas are from the same field of GCs. Data represent the means ± SD of GCs and somas from five to eight mice. Statistical significance was determined by one-way ANOVA followed by Dunnett’s multiple comparisons test. YFP, yellow fluorescent protein; CFP, cyan fluorescent protein. (**D**) Quantification of cytosolic ATP in GCs of primary motoneurons transfected with the cytosolic ATeam sensor in galactose medium. Data represent the means ± SD of GCs from five mice. Statistical significance was determined by one-way ANOVA followed by Dunnett’s multiple comparisons test. (**E**) GCs stained with Tyr-TUB (green) and Actin (Phalloidin, red) of motoneurons grown in medium with or without uridine supplementation. Scale bar, 10 μm. (**F**) Quantification of the size of GCs in experiments as in (E). Data represent the means ± SD of five to seven independent cultures (27 to 66 GCs per culture). Statistical significance was determined by one-way ANOVA followed by Dunn’s multiple comparisons test.

GCs dynamically remodel the cytoskeleton in response to attractive and repulsive chemical guidance cues. This process is supported by cycles of de- and re-polymerization of actin and microtubules that require boosts of energy. Because CLUH deficiency impairs mitochondrial respiration (*16*, *17*, *19*), insufficient adenosine 5′-triphosphate (ATP) production from mitochondria may underlie the distal defects of NKO motoneurons. We measured mitochondrial ATP in soma and GCs using the ratiometric sensor ATeam targeted to mitochondria (*30*). ATP levels were decreased in mitochondria of NKO GCs compared to WT but were unaffected in soma mitochondria (Fig. 5, A to C). Blockage of the ATP synthase by oligomycin reduced the levels of mitochondrial ATP in WT but decreased it only slightly in NKO GCs (Fig. 5C). To test whether reduced ATP production from mitochondria affects the cytosolic ATP levels, we transfected a cytosolic sensor and performed the analysis in presence of galactose (25 mM) instead of glucose to limit anaerobic respiration, which is preferentially used by neurons, and promote oxidative metabolism (*31*). Under these experimental conditions, ATP levels were decreased in NKO GCs (Fig. 5D). Moreover, the acute inhibition of mitochondrial ATP synthase by oligomycin induced the collapse of WT GCs to a greater extent than NKO GCs (fig. S6, A and B, and movie S4). Thus, CLUH maintains mitochondrial ATP levels in GCs.

While ATP production by GC mitochondria may be fundamental under conditions of glucose limitation, it is known that glycolysis metabolically supports distal axons and GC dynamics (*32*, *33*). However, a dysfunctional respiratory chain can also affect the nucleotide pool because the mitochondrial rate-limiting enzyme in pyrimidine synthesis, dihydroorotate dehydrogenase (DHODH), uses coenzyme Q as electron acceptor (*34*). For this reason, respiratory deficient cells cannot grow without uridine supplementation (*35*, *36*). Uridine not only is a precursor of RNA but also is required for the synthesis of phospholipids and therefore supports membrane growth. Moreover, it can be converted in ribose-1-phosphate that enter glycolysis to fuel ATP production (*37*). We therefore tested whether uridine supplementation could recover the GC phenotype in NKO axons. Notably, the area of NKO GCs increased in presence of uridine (Fig. 5, E and F), supporting the hypothesis that a complex metabolic deficit of distal mitochondria, and not simply ATP defects, contributes to collapsed GCs.

### CLUH controls the abundance of target mRNAs in axons

**Fig. 6.**
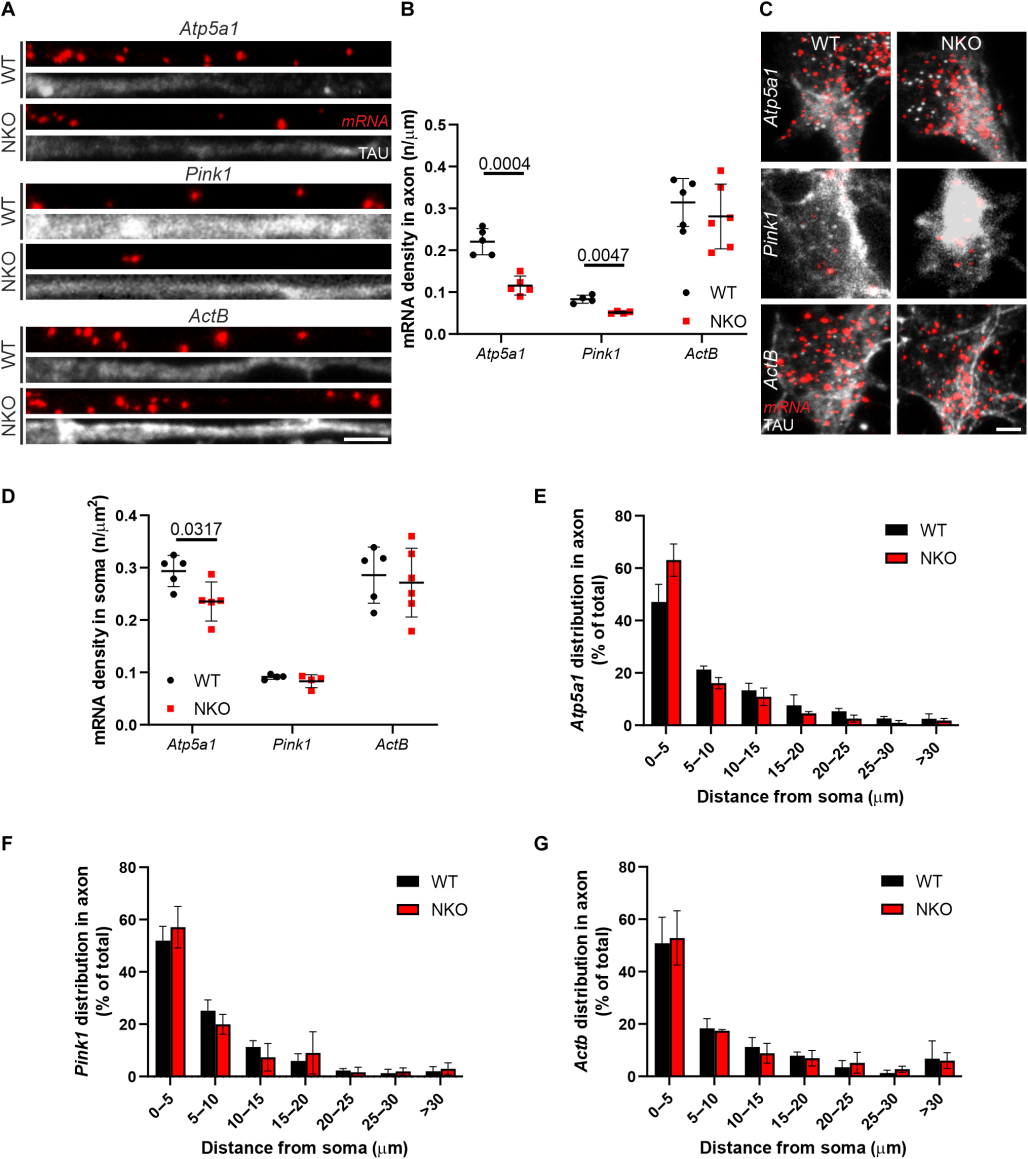
Lack of CLUH depletes axons and somas of CLUH target mRNAs. (**A**) RNAscope of *Atp5a1*, *Pink1*, and *ActB* mRNAs (red) in axons of primary spinal motoneurons (DIV 6) stained for TAU (gray). Scale bar, 5 μm. (**B**) Quantification of the abundance of *Atp5a1*, *Pink1*, and *ActB* mRNAs in axons in experiments as in (A). Data represent the means ± SD of four to six mice (26 to 57 neurons per mouse). Statistical significance was determined by Welch’s *t* test. (**C**) RNAscope of *Atp5a1*, *Pink1*, and *ActB* mRNAs (red) in somas of primary spinal motoneurons (DIV 6) stained for TAU (gray). Scale bar, 5 μm. (**D**) Quantification of the abundance of *Atp5a1*, *Pink1*, and *ActB* mRNAs in somas in experiments as in (C). Data represent the means ± SD of four to six mice (26 to 57 neurons per mouse). Statistical significance was determined by Mann-Whitney and Welch’s *t* test. (**E** to **G**) Distribution of *Atp5a1* (E), *Pink1* (F), and *ActB* (G) mRNAs particles along axons of primary motoneurons in experiments as shown in (A). Data represent the means ± SD of three to five cultures (72 to 653 mRNA dots per culture).

CLUH may affect mitochondrial respiration and metabolism by regulating target mRNAs at different levels. We first checked whether the abundance of CLUH target mRNA molecules was similarly affected in cell bodies and axons of primary motoneurons and selected *Atp5a1* and *Pink1* for further analysis by fluorescence in situ hybridization. *Atp5a1* mRNA molecules were reduced in both NKO cell bodies and axons, but this reduction was more pronounced in axons, in agreement with the distal ATP defect (Fig. 6, A to D). Consistently, *Pink1* mRNA molecules were also decreased in the axon in the absence of CLUH. *ActB* mRNA, which is not bound by CLUH, was not altered in NKO somas and axons (Fig. 6, A to D). Notably, the distribution of mRNA molecules for all transcripts analyzed was similar in WT and NKO axons (Fig. 6, E to G).

One possible explanation for the previous findings is that mRNAs are less transported into axons when not bound by CLUH. To directly visualize trafficking of mRNAs in WT and NKO motoneurons, we used the bacteriophage-derived MS2 tether system. The CLUH mRNA targets *Atp5a1* and *Mdh2* and the *Actb* mRNA as a control were tagged after the 3′ untranslated region (3′UTR) with 24 repeats of the MS2 stem-loops and expressed in neurons together with a reporter construct encoding the MS2 coat protein (MCP) fused with a Halo tag for fluorescent detection (fig. S7A). For analysis, neurons showing axonal mRNA particles were selected. The time in motion of *Atp5a1*-, *Mdh2*-, and *Actb*-tagged mRNAs was similar in WT and NKO axons (fig. S7, B and C). Furthermore, we did not find significant changes in the type of movement of the mRNA particles, assessed either by lateral displacement over the whole track (directed, oscillatory, or stationary) (fig. S7D) or by the mean square displacement (MSD) in the first 25% of the track (active, diffusive, or confined) (fig. S7E). Thus, the reduced axonal localization of CLUH target mRNAs is likely caused by a decrease of mRNA stability rather than a trafficking defect along the axons.

### Compartmentalized proteomics reveals mitochondrial and translational signatures in NKO axons

**Fig. 7.**
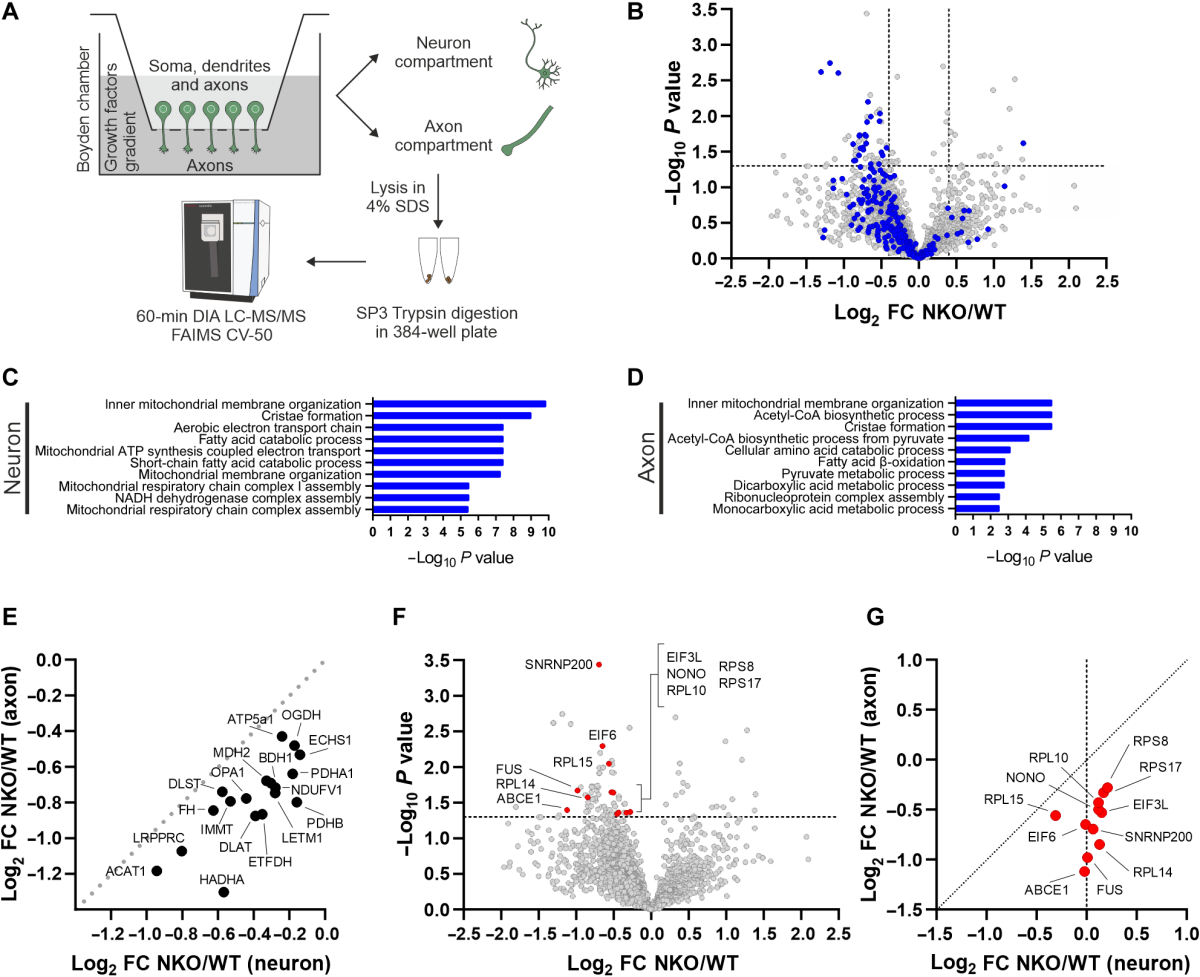
Mitochondrial proteins and translation components are depleted in NKO axons. (**A**) Experimental setup to isolate axons of spinal motoneurons using Boyden chambers. A gradient of growth factors was applied from the lower axonal compartment to the upper compartment containing soma, dendrites, and axons. Axons were scraped and lysed in a 4% SDS solution; immediately afterward, the upper compartment was lysed in the same solution. Samples were then digested in SP3 trypsin buffer and subsequently analyzed using DIA LC-MS/MS FAIMS at compensation voltage (CV) of −50. Figure created with Biorender.com. (**B**) Volcano plot of protein changes in NKO versus WT axons. Mitochondrial proteins are highlighted in blue. (**C** and **D**) GOBP analysis of down-regulated proteins in the neuron (C) and axon (D) compartments of primary motoneurons grown in Boyden chambers. Analysis was done using the EnrichR webtool. NADH, reduced form of nicotinamide adenine dinucleotide (oxidized form); CoA, coenzyme A. (**E**) Correlation of the fold change of proteins encoded by CLUH mRNA targets in NKO versus WT axon and neuron compartments. Only proteins measured in axons are indicated. (**F**) Volcano plot of proteins altered in NKO versus WT axons. Proteins related to translation or RNA binding are highlighted in red. (**G**) Correlation of the fold changes of proteins highlighted in (F) in NKO versus WT in axon and neuron compartments.

To further unravel the underlying cause for the axonal phenotype, we examined whether CLUH depletion affects the proteome differently depending on the neuronal compartments. To this end, we isolated the central and the peripheral portion of neurons using modified Boyden chambers (*38*) and performed proteomics analysis (Fig. 7A). Motoneurons were grown on the porous membrane in the upper compartment of the chamber (from now on named neuron compartment) and only neurites could pass into the bottom compartment. Because embryonic motoneurons have very short dendrites, the bottom compartment is highly enriched of axons (from now on named axon compartment; fig. S8A). Primary motoneurons were isolated from different embryos in independent experiments and grown in Boyden chambers for 6 days before collecting the two compartments. Given the paucity of material of the axonal compartment, two samples of the same genotype and neuronal culture were pooled before mass spectrometry (MS) analysis. The proteomics of the axonal compartment relative to the neuron compartment revealed an enrichment of axonal markers and a depletion of proteins located in the nucleus, endoplasmic reticulum, and Golgi (fig. S8B), confirming the quality of the separation. Because principal components analysis showed a batch effect associated with the day of neuronal isolation and the litter (fig. S8, C and D), data were transformed to log_2_ fold changes (log_2_FCs) of NKO versus WT samples for each individual litter and neuronal isolation followed by a one-sample *t* test. Proteins were included in the analysis if quantified in at least three of the seven NKO-WT comparisons. Accordingly, 2800 proteins were identified in the axonal and 6453 in the whole neuronal compartment (table S2). In the NKO neuron–containing fractions, 52 proteins were increased (log_2_FC > 0.4; *P* < 0.05), and 117 were decreased (log_2_FC < −0.4; *P* < 0.05) relative to WT. In the NKO axonal fractions, 14 proteins were up-regulated (log_2_FC > 0.4), and 60 proteins were down-regulated (log_2_FC < −0.4) (Fig. 7B). In both compartments, gene ontology analysis did not identify any specific up-regulated pathway but showed an overrepresentation of mitochondrially related terms among the down-regulated proteins (Fig. 7, C and D, and fig. S8E). Thus, mitochondrial proteins were decreased centrally and distally in the absence of CLUH. We observed a significant overlap between mitochondrial proteins whose steady-state level was decreased and those encoded by CLUH mRNA targets (fig. S8F) (*11*). Of note, these were more decreased in axons with respect to the whole neurons (Fig. 7E). These proteins included components of the respiratory chain, TCA cycle, β-oxidation, and other metabolic enzymes.

Besides mitochondrial proteins, proteins reduced in NKO axons included other RBPs [fused in sarcoma (FUS) and non-POU domain containing octamer binding], the splicing factor SNRNP200, crucial components for the initiation and regulation of translation (ABCE1, and the eukaryotic initiation factors 3 subunit L and 6), and several ribosomal subunits (ribosomal proteins L10, L14, L15, S8, and S15) (Fig. 7F). We confirmed these results by measuring the decreased intensity of the fluorescence of ABCE1, RPL14, and RPS8 in NKO axons (fig. S9, A and B). In contrast to mitochondrial proteins, these translational components were unchanged in the compartment containing the cell bodies (Fig. 7G). Thus, while the mitochondrial proteomic signature is common to neuronal soma and axons, albeit more pronounced in axons, the translational signature was restricted to axons.

### Axonal translation and *Atp5a1* mRNA defects induced by loss of CLUH are rescued by ABCE1 overexpression

**Fig. 8.**
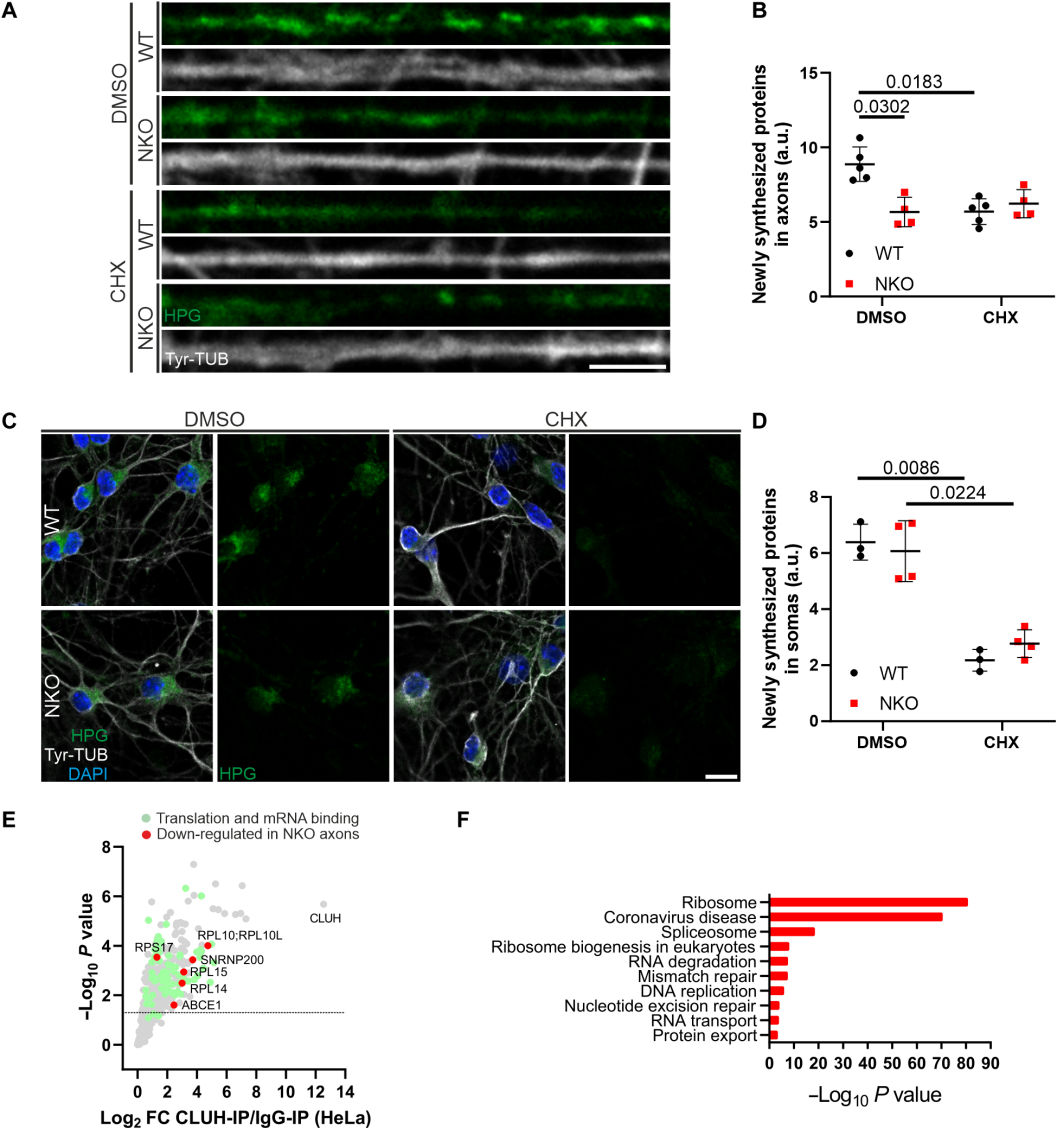
CLUH absence impairs translation in axons but not in somas. (**A** and **B**) Single confocal planes (A) and quantification (B) of newly synthesized proteins (HPG, green) revealed by the FUNCAT assay in WT and NKO axons stained with Tyr-TUB (gray). Data represent the means ± SD of four to five mice (27 to 71 axons per culture). Statistical significance was determined by one-way ANOVA followed by Dunnett’s multiple comparisons test. CHX, cycloheximide; HPG, l-homopropargylglycine; Tyr-TUB, tyrosinated tubulin. Scale bar, 5 μm. (**C** and **D**) Primary motoneurons (C) and quantification (D) of newly synthesized proteins (HPG, green) in WT and NKO somas stained with Tyr-TUB (gray). Data represent the means ± SD of three to four mice (49 to 100 somas per culture). Statistical significance was determined by one-way ANOVA followed by Dunnett’s multiple comparisons test. Scale bar, 20 μm. (**E**) Volcano plot of the interactome of CLUH in HeLa cells. Green dots denote proteins involved in translation and RNA binding; red dots denote interactors of CLUH decreased in NKO axons. (**F**) Kyoto Encyclopedia of Genes and Genomes pathway analysis of the interactome of CLUH in HeLa cells. Analysis was done using the EnrichR webtool.

The previous findings prompted us to examine whether CLUH is required to preserve axonal translation. To this end, we used fluorescent noncanonical amino acid tagging (FUNCAT), which uses the incorporation of the methionine analog homopropargylglycine (HPG) in nascent proteins and the subsequent labeling via click reaction with a fluorescent azide. These experiments established that defects of translational components were reflected in reduced protein synthesis in NKO axons (Fig. 8, A and B) but not in somas (Fig. 8, C and D). It is known that mitochondrial proteins are highly translated in axons (*10*); however, it is still unexpected that lack of CLUH leads to a general defect of translation in axons. This result prompted us to investigate the underlying molecular mechanisms. We therefore immunoprecipitated endogenous CLUH in HeLa cells followed by MS to reveal its interactome. We found that several ribosomal components and translational regulators, including those depleted in NKO axons, were significantly enriched in the CLUH immunoprecipitation (IP) versus the control IP, demonstrating a link between CLUH and the translational machinery (Fig. 8, E and F, and table S3).

**Fig. 9.**
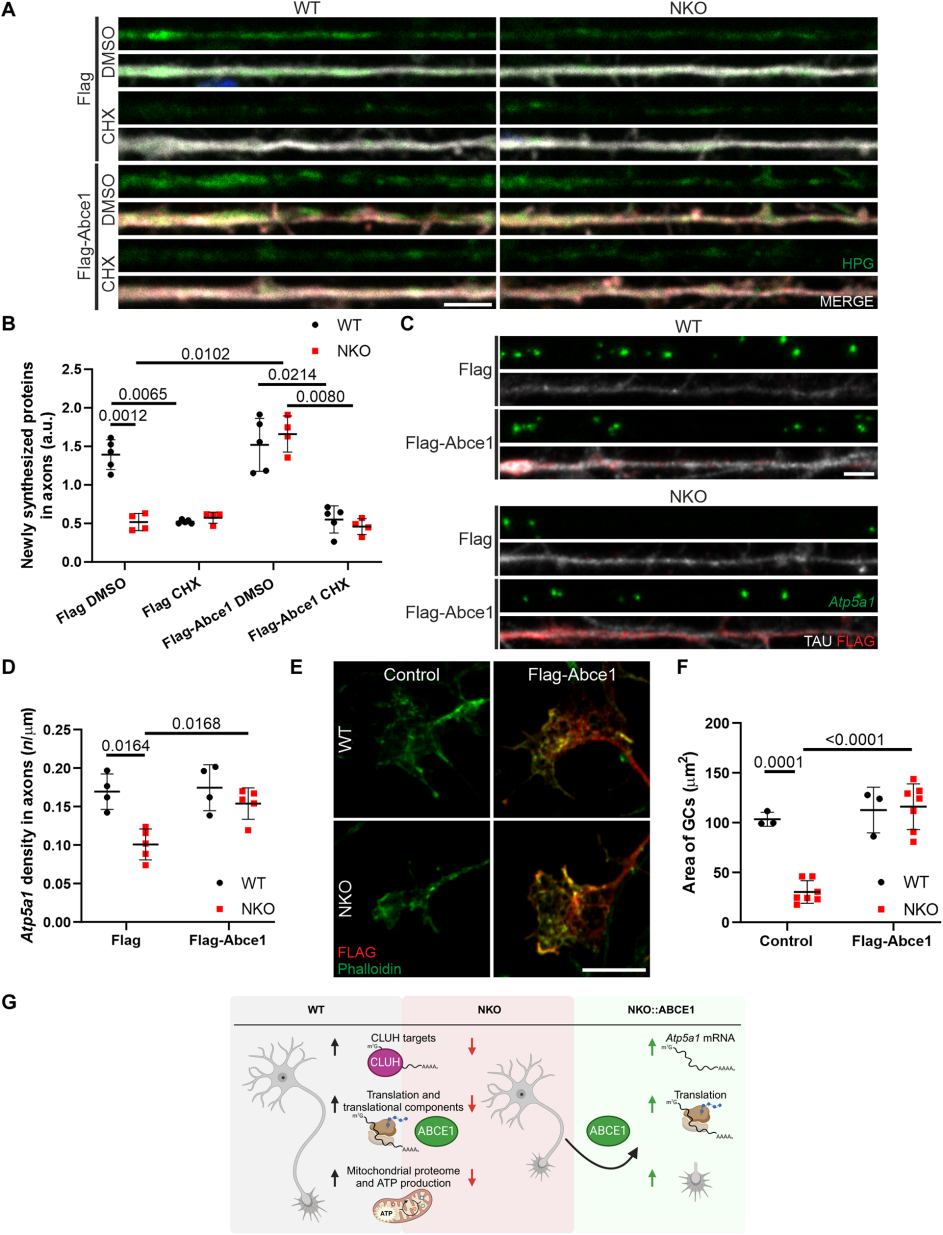
ABCE1 restores translation, *Atp5a1* mRNA, and GCs size in NKO axons. (**A** and **B**) Single confocal planes (A) and quantification (B) of newly synthesized proteins (HPG, green) revealed by the FUNCAT assay in WT and NKO axons transfected with Flag or Flag-Abce1 (red) and stained with TAU (gray). The MERGE panels show HPG, Flag or Flag-Abce1, TAU, and DAPI (blue). Data represent the means ± SD of four to five mice (10 to 22 axons per culture). Statistical significance was determined by one-way ANOVA followed by Dunnett’s multiple comparisons test. Scale bar, 5 μm. (**C**) RNAscope of *Atp5a1* (green) in axons of primary spinal motoneurons (DIV 6) transfected with Flag or Flag-Abce1 (red) and stained for TAU (gray). Scale bar, 5 μm. (**D**) Quantification of the abundance of *Atp5a1* mRNA in axons in experiments as in (C). Data represent the means ± SD of four to five mice (18 to 39 neurons per mouse). Statistical significance was determined by one-way ANOVA followed by Dunnett’s multiple comparisons test. (**E** and **F**) Single confocal planes (E) and quantification (F) of the GCs area of GCs of control and Flag-Abce1 (red) transfected motoneurons stained for Actin (Phalloidin, green). Data represent the means ± SD of three to seven cultures (17 to 34 GCs per culture). Statistical significance was determined by one-way ANOVA followed by Dunnett’s multiple comparisons test. Scale bar, 10 μm. (**G**) Scheme depicting phenotypes observed in axons of spinal motoneurons lacking CLUH. Lack of CLUH is associated to reduced levels of mRNA targets, impairment of axonal translation, and mitochondrial dysfunction characterized by decreased ATP production and proteome abnormalities. The overexpression of ABCE1 in NKO neurons restores the levels of *Atp5a1* mRNA, axonal translation, and the size of GCs. Figure created with Biorender.com.

If the lack of essential translational components distally underlies the axonal translation and GC defects in NKO motoneurons, then the expression of depleted proteins could be beneficial. ABCE1 caught our attention because it is the most down-regulated protein related to translation in axons (Fig. 7, F and G) and was enriched in the CLUH IP (Fig. 8E). ABCE1 is an essential regulator of initiation and termination of translation, recycling, and quality control of ribosomes (*39*). Transfected FLAG-tagged ABCE1 localizes to GCs, with a pattern very similar to that of overexpressed CLUH (fig. S9C). We therefore expressed ABCE1 in NKO neurons and monitored axonal translation. Notably, expression of ABCE1 restored axonal protein synthesis in NKO axons (Fig. 9, A and B). Given this result, we asked whether restoring ABCE1 in axons can also protect the CLUH target mRNAs from degradation. *Atp5a1* mRNA molecules in axons were significantly increased in NKO axons upon ABCE1 overexpression (Fig. 9, C and D). This molecular rescue by ABCE1 was accompanied by a phenotypic rescue at the level of GC size (Fig. 9, E and F). In conclusion, we identify a critical role of CLUH in axons to maintain not only target mRNAs and mitochondrial function but also translational capacity and uncover ABCE1 as a molecular factor acting downstream of CLUH in the pathogenic cascade (Fig. 9G).

## DISCUSSION

Highly polarized neurons such as spinal motoneurons depend on mRNA transport and localized translation for development and survival and often degenerate in neurological conditions caused by mutations in RBPs implicated in these processes (*40*). Here, we demonstrate a functional role of CLUH for development and maintenance of peripheral axonal compartments. We link this phenotype to impaired mitochondrial metabolism, owing to depletion of mitochondrial proteins encoded by the CLUH target mRNAs. Mechanistically, we propose a role of CLUH in translational regulation of its target mRNAs.

CLUH ablation in murine neural precursors in vivo mainly results in axonal degeneration, with progressive motor dysfunction and loss of axons in the sciatic nerve. In vitro, CLUH is essential for supporting axonal growth of spinal motoneurons, indicating a developmental role. Consistently, NKO embryos displayed a delay of hindlimb innervation; however, this developmental defect appeared to be compensated at later times. An early axonal growth defect, manifesting as neurodegeneration in adult mice, has been described in models of Huntington’s disease, and the contribution of a developmental component to neurodegeneration has been proposed in other classical neurodegenerative conditions (*41*, *42*). Various mechanisms, such as circuit remodeling and metabolic interactions with surrounding glial cells, may account for the initial rescue of developmental abnormalities in vivo but could render neurons prone to degeneration later in life.

By comparing the effect of CLUH depletion in neuronal somas versus axons, we found that lack of CLUH affects the abundance of targets mRNAs and their encoded mitochondrial proteins in neurites more than in the neuronal cell bodies, explaining the axonal-restricted pathology and highlighting the vulnerability of axons to mitochondrial dysfunction. In agreement with the large numbers of CLUH mRNA targets, NKO neurons were depleted of several mitochondrial proteins, implicated in different metabolic pathways. We found that the ability of GC mitochondria to maintain ATP levels was hampered, consistent with a defect of the respiratory chain. Moreover, we found that NKO neurons displayed uridine auxotrophy, a phenotype so far observed in proliferating cells with OXPHOS defects. The mitochondrial enzyme DHODH that converts dihydroorotate in orotate, a step of pyrimidine biosynthesis, uses coenzyme Q as electron acceptor and is therefore dependent on a functional respiratory chain (*34*). Notably, DHODH is a putative CLUH target mRNA (*11*). Uridine is a precursors of RNA, phospholipids, and ribose-1-phosphate, which can be converted to glycolytic intermediates (*37*). Uridine supplementation may therefore compensate a mitochondrial dysfunction in postmitotic neurons by promoting membrane biogenesis and even fueling ATP production in the cytosol. Previous studies have shown a beneficial role of uridine to support neuronal survival under conditions of glucose deprivation (*43*).

Our data support the hypothesis that CLUH deficiency leads to reduced axonal levels of its mRNA targets due to destabilization rather than a transport defect. mRNA trafficking assays, using *Atp5a1* and *Mdh2* as representative targets, showed that the tagged mRNAs traveled along WT and NKO axons for the same time and with similar features in respect to directed versus oscillatory movement or confined localization. In further agreement with our conclusion is the observation of a decreased axonal abundance of the *Pink1* mRNA, which is known to be transported via binding to synaptojanin 2 and synaptojanin 2 binding protein (*5*). The stability of mRNAs is per se an important determinant of neurite localization (*44*). Notably, CLUH target mRNAs are depleted of destabilizing factors, increasing the likelihood that they reach the peripheral regions of a neuron (*44*). Two hundred seven of the 229 putative CLUH targets contain less than nine A-U–rich elements in the 3′UTR and only 26 of the 229 are m^6^A (*N*^6^-methyladenosine)–enriched (*44*). While CLUH may simply be another stabilizing factor during the mRNA life cycle, several pieces of evidence suggest a role of CLUH in translational regulation, causing a secondary mRNA degradation. First, NKO axons were characterized by depletion of ribosomal components and translational regulators and by impaired translation. Although the pronounced translational defects of NKO axons seem unexpected, mitochondrial proteins are highly translated in axons (*10*). Moreover, at least in dendrites, mitochondria act as reservoirs to provide the energy required for localized translation (*45*); thus, the mitochondrial dysfunction caused by loss of CLUH may exacerbate defects also in the translation of mRNAs that are not direct CLUH targets. Second, the CLUH interactome in HeLa cells indicated proximity to the translational machinery. Previous studies found CLUH in the vicinity of mitochondrial proteins before import and of mitochondrial-associated ribosomes, substantiating a role of CLUH during some step of translation of its targets (*46*–*48*). Third, the reexpression of ABCE1 in NKO axons was sufficient to rescue the levels of *Atp5a1* mRNA, the translational defect, and the GC size of NKO axons.

Regulation of axonal translation occurs mainly at the level of initiation, and failure to activate translation at the right time and place or to recycle ribosomes after a translation cycle could be especially detrimental. ABCE1 is a fundamental player coupling translation termination and the splitting of the two ribosomal subunits to translation initiation (*39*). By allowing the translation cycle to resume, ABCE1 counteracts the removal of ribosomes and associated mRNAs by ribophagy (*49*). Thus, if lack of CLUH causes a defect in translation initiation or induces translation stalling, triggering downstream ribosomal quality control pathways, then ABCE1 overexpression could be beneficial. Such a model would be consistent with previous data in primary hepatocytes, where CLUH target mRNAs were found in G3BP1-positive granules that were delivered to the lysosomes upon starvation faster in the absence of CLUH (*50*).

In *Drosophila*, ectopic ABCE1 expression reverses the mitochondrial aggregation caused by *Pink1* down-regulation in the muscle. In this context, stalled ribosomes on damaged mitochondria lead to polyubiquitination of ABCE1 by CCR4-NOT transcription complex subunit 4. ABCE1 then acts as a signal to initiate mitophagy by recruiting autophagic receptors to the mitochondria (*51*). In agreement with this role, ABCE1 overexpression also inhibits the accumulation of longer forms of respiratory chain proteins generated by co-translational extension upon mitochondrial dysfunction (*52*). Notably, the *Drosophila* *Cluh* ortholog *clueless* interacts genetically with *Pink1* (*53*) and suppresses phenotypes of *Pink1* mutants when overexpressed (*53*, *54*). Thus, PINK1 and CLUH may be involved in parallel pathways that initiate mitophagy upon different types of translational defects on the mitochondrial surface. We previously found that mitophagy was affected in cells lacking CLUH (*50*). More experiments are needed to understand the role of CLUH in translation and whether ribophagy is initiated upon lack of CLUH, depleting axons of mRNAs for mitochondrial proteins and of many ribosomal components, including ABCE1, causing a downstream broad translation defect. Whether CLUH acts at the mitochondrial surface remains to be determined.

Together, we identify CLUH as a crucial player for the expression of mitochondrial proteins in axons and show the physiological role of CLUH to prevent peripheral neuropathy. The central and spinal motoneurons with long axons that show pathology upon CLUH depletion are affected also upon mutations in other RPBs, such as survival motor neuron, FUS, and transactive response DNA binding protein-43 (*55*), which cause motoneuron disease and lead to defects in axonal translation and mitochondrial dysfunction. It will be interesting to explore whether dysfunctional CLUH plays any role in the pathogenesis of these neurodegenerative diseases.

## MATERIALS AND METHODS

### Mouse lines

All animal procedures were carried out in accordance to the European (EU directive 86/609/EEC), national (Tierschutzgesetz), and institutional guidelines and were approved by local authorities (Landesamt für Natur, Umwelt und Vebraucherschutz Nordrhein-Westfalen, Germany). Animals were maintained in the Cologne Excellence Cluster on Cellular Stress Responses in Aging-Associated Diseases (CECAD) Research Center, University of Cologne, Germany, in individually ventilated cages at 22°C (± 2°C) and a relative humidity of 55% (± 5%) under 12-hour light cycle on sterilized bedding (Aspen wood, Abedd, Germany) and with access to sterilized commercial pelleted diet (Ssniff Spezialdiäten GmbH) and acidified water ad libitum. Previously described *Cluh*^fl/fl^ mice (*16*) were crossed with a nestin-Cre transgenic mice line (*23*) to generate a neural *Cluh*^fl/fl^ Cre^wt/tg^ (NKO) mice. Mice were kept on a pure C57/BL6N. *Cluh*^fl/fl^ Cre^wt/wt^ (WT) and *Cluh*^wt/wt^ Cre^wt/tg^ (Cre) mice were used as controls. Mice used for behavioral tests were analyzed by sex, and tissues for histology and embryos were used independent of the sex. Animals were killed by CO_2_ inhalation to collect organs, and pregnant females were killed by cervical dislocation to collect embryos. When required, mice were anesthetized with 20 mg of xylazine/100 mg of ketamine per kilogram of body weight and intracardially perfused with 4% paraformaldehyde (PFA; Sigma-Aldrich) in phosphate-buffered saline (PBS).

### Walking beam and rotarod tests

Animals were trained to cross a 90-cm-long wide beam for 2 days. The training included the acclimatization from a 3-cm- to a 1-cm-wide beam and 10 min of rest between the two sessions. On the third day, animals were tested three times to cross the 1-cm-wide beam. The number of slips and time to cross the beam were quantified. After 2 days of rest, mice were tested using a RotaRod apparatus (TSE Systems). Mice were trained for 5 min to acclimatize on the accelerating rod without acceleration. The latency to fall from the apparatus was tested three times at 4 rpm and a constant acceleration of 7.2 rpm/min, with a resting period of 15 min between each trial.

### Nerve conduction

CMAP amplitudes were recorded using a PowerLab single acquisition setup (ADInstruments, Grand Junction). After anesthesia, the sciatic nerves were stimulated using needle electrodes, and the amplitude of the CMAP was recorded using recording needle electrodes into the hind paw as previously described (*56*).

### RNA extraction and transcriptomics

Freshly dissociated sciatic nerves from 5-month-old mice were snap-frozen in liquid nitrogen and stored at −80°C. The RNA from sciatic nerves was isolated using the RNeasy Microkit (QIAGEN) according to the instructions of the manufacturer. Samples were processed by the Cologne Center for Genomics. Because low amount of input material, pre-amplification using the Ovation RNASeq System V2 was performed. Total RNA was used for first-strand cDNA synthesis, using both poly(T) and random primers, followed by second-strand synthesis and isothermal strand-displacement amplification. For library preparation, the Illumina Nextera XT DNA sample preparation protocol was used, with 1 ng of cDNA input. After validation (Agilent 2200 TapeStation) and quantification (Invitrogen Qubit System), all six transcriptome libraries were pooled. The pool was quantified by using the Peqlab KAPA Library Quantification Kit and the Applied Biosystems 7900HT Sequence Detection System. The pool was sequenced on an Illumina NovaSeq6000 sequencing instrument with a PE100 protocol. RNA sequencing (RNA-seq) was performed with a directional protocol. Quality control, trimming, and alignment were performed using the nf-core 56 RNA-seq pipeline (v3.0) (https://doi.org/10.5281/ZENODO.1400710). The reference genome sequence and transcript annotation used were *Mus musculus* genome GRCm39 from Ensembl version 103. Differential expression was analyzed in R version 4.1.2 (www.R-project.org/) with DESeq2 v1.34.0 (*57*) to make pairwise comparisons between groups. Log fold change shrinkage estimation was performed with ashr (*58*). Genes were considered as candidates to be differentially expressed if they had a minimum coverage of 10 reads in six or more samples from each pairwise comparison. Genes were significant if they met the following criteria: *P* < 0.05, *q* < 0.05, and fold change < −0.50 or > 0.50. Gene ontology analysis of significantly enriched genes was performed using the EnrichR webtool (*59*).

### Histology

For free-floating staining, 30-μm sections of fixed spinal cords were cut using a vibratome (VS1000, Leica), permeabilized and blocked with 0.4% Triton X-100 (Sigma-Aldrich) and 10% fetal bovine serum (FBS; Sigma-Aldrich) for 1 hour, and incubated with polyclonal goat anti-CHAT (AB144P, Millipore) in 5% FBS overnight (ON). Then, samples were incubated with donkey anti-goat immunoglobulin G (IgG) Alexa Fluor 488 (A11055, Thermo Fisher Scientific) for 2 hours. Nuclei were counterstained using 4′,6-diamidino-2-phenylindole (DAPI) and mounted using ProLong Gold Antifade Reagent (Cell Signaling Technology).

For NMJ staining, freshly dissected tibialis anterior muscles were fixed with 4% PFA/PBS ON; cut into 250-μm sections using a vibratome, permeabilized and blocked in blocking buffer [3% bovine serum albumin (Sigma-Aldrich), 5% goat serum (Euroclone), and 0.5% Triton X-100] for 2 hours, incubated with primary antibody rabbit monoclonal anti–neurofilament N (C28E10, Cell Signaling Technology) in 1% blocking buffer at 4°C ON and then with goat anti-rabbit IgG Alexa Fluor 647 (A21245, Thermo Fisher Scientific) and α-Bungarotoxin Alexa Fluor 488 (B13422, Thermo Fisher Scientific) for 2 hours. Samples were mounted using FluorSave reagent (Calbiochem). The innervation of NMJs was classified in full, partial, and vacant, as previously described (*60*).

Hindlimbs of E13.5 embryos were stained using a modified protocol (*61*). Embryos were fixed in 4% PFA ON and permeabilized with 0.2% Triton X-100/20% dimethyl sulfoxide (DMSO) at 37°C ON and then in 0.1% Tween 20/0.1% Triton X-100/0.1% deoxycholate/0.1% NP-40/20% DMSO at 37°C ON. Embryos were blocked in 0.2% Triton X-100/10% DMSO/6% goat serum at 37°C for 2 days and then incubated with mouse monoclonal anti–neurofilament M primary antibody (2H3, Developmental Studies Hybridoma Bank) in 0.2% Tween 20 with heparin (10 μg/ml)/5% DMSO/3% goat serum at 37°C for 3 days, followed by the incubation with secondary antibody goat anti-mouse IgG Alexa Fluor 488 at 37°C for 2 days. Images were acquired using the HC PL APO CS2 20×/0.75 DRY and HC PL APO CS 40×/0.85 DRY objectives of an SP8 confocal microscope (Leica).

### Semithin sections and electron microscopy

Sciatic nerves from perfused animals were postfixed in 2% glutaraldehyde (Sigma-Aldrich) in 0.1 M cacodylate buffer (Sigma-Aldrich). Sciatic nerves were cut distally and stained with 1% toluidine blue. Semithin sections were imaged using the Plan-Apochromat 20×/0.8 objective of an Axio-Imager M2 microscope equipped with Apotome 2 (Zeiss). The number of axons was automatically segmented and quantified using the Trainable Weeka Segmentation plugin of ImageJ (National Institutes of Health, Bethesda). Degenerating axons were manually quantified as dark spots and normalized by the surface of the cross section. For ultrastructural studies, sciatic nerves were embedded in Epon and imaged as previously described (*16*), using a transmission electron microscope (JEOL JEM2100PLUS) provided with a GATAN OneView camera. The morphology of mitochondria was manually assessed by an experimenter blind to the experiment.

### DNA constructs

Mito–green fluorescent protein (*62*), pcDNA-AT1.03 and pcDNA-mitoAT1.03 (*30*), and pcDNA3.1-Flag-*Abce1* (*63*) (provided by R. S. Hedge) were previously described. Full-length human *CLUH* and mouse *Cluh* coding sequences cDNA were cloned in pcDNA3 and pmCherry-N1 (Clontech), respectively, using Hind III and Eco RI restriction sites. For mRNA trafficking, target mRNAs were tagged with 24 repeats of the MS2V5 sequence behind their 3′UTR. To achieve these constructs, the HaloTag-*bActin*CDS-*bActin*UTR-MS2V5 plasmid (Addgene, no. 102718) (*64*) was used as a basis for the cloning. An initial restriction digest using Not I and Age I removed all tags and the *ActB* mRNA, leaving only the MS2V5 sequence. *ActB*, *Mdh2*, and *Atp5a1* mRNA were cloned back into the backbone by Gibson Assembly, using the Addgene plasmid no. 102718 as a template for *ActB* and mouse cDNA as a template for *Mdh2* (ENSMUST00000019323) and *Atp5a1* (ENSMUST0000002649). For the reporter plasmid, NLS-HA-stdMCP-stdHalo (Addgene, no. 104999) (*65*) was used.

### Cell lines and cell culture

Immortalized mouse embryonic fibroblasts (MEFs) were previously described (*11*). MEFs were electroporated using P3 primary cell 4D-nucleofector X kit L (V4XP-3024, Amaxa) using Nucleofector I (Amaxa). Spinal cords were dissected from E13.5 embryos, and primary motoneurons were isolated as previously described (*66*). Motoneurons were plated on coverslips coated with poly-d-lysine (20 μg/ml; Sigma-Aldrich) and laminin (0.1 μg/ml; Sigma-Aldrich) at a density of 15,000 cells/cm^2^ and grown in Neurobasal medium (Thermo Fisher Scientific) supplemented with 2% B-27 (Thermo Fisher Scientific), 1% glutamine (Thermo Fisher Scientific), 1% penicillin/streptomycin (Thermo Fisher Scientific), amphotericin B (250 μg/ml; Promocell), 1 μM cytosine arabinoside (Sigma-Aldrich), brain-derived neurotrophic factor (BDNF; 10 ng/ml; Peprotech), ciliary neurotrophic factor (CNTF; 10 ng/ml; Peprotech), and glia cell line–derived neurotrophic factor (GDNF; 10 ng/ml; Peprotech). The medium was supplemented with uridine (50 μg/ml) where indicated. Cells were cultured in a humified incubator with 5% CO_2_ at 37°C. For neurons grown in Boyden chambers (1.0-μm pore size, Falcon), the medium contained in the bottom compartment was supplemented with five times the concentration of BDNF, CNTF, and GDNF. Neurons were transfected with Lipofectamine 2000 (Thermo Fisher Scientific) according to (*67*).

### Immunocytofluorescence

Cells were fixed with 4% paraformaldehyde (PFA; Sigma-Aldrich) for 20 min, permeabilized with 0.1% Triton X-100 for 10 min, blocked with 10% goat serum for 1 hour, and incubated with primary antibodies in 1% goat serum ON. The following antibodies were used: monoclonal rabbit anti-ABCE1 (ab32270, Abcam), polyclonal rabbit anti-CLUH (NB100-93306, Novus Biologicals), monoclonal mouse anti-Cherry (677702, BioLegend), monoclonal rat anti-CLASP2 (MAB9738, Abnova), mouse monoclonal anti-FLAG (F3165, Sigma-Aldrich), polyclonal rabbit anti-IQGAP1 (2217-1-AP, Proteintech), polyclonal rabbit anti-MAP2 (4542, Cell Signaling Technology), polyclonal rabbit anti-RFP (600-401-379S, Rockland), polyclonal rabbit anti-RPL14 (14991-1-AP, Proteintech), polyclonal rabbit anti-RPS8 (ab201454, Abcam), polyclonal rabbit anti–synapsin 1/2 (106002, Synaptic Systems), monoclonal mouse anti-TAU (sc-390476, Santa Cruz Biotechnology), polyclonal rabbit anti-TOM20 (sc-114115, Santa Cruz Biotechnology), monoclonal mouse anti–β-tubulin III (T8660, Sigma-Aldrich), monoclonal mouse anti-acetylated tubulin (T6793, Sigma-Aldrich), and monoclonal mouse anti-tyrosinated tubulin (T9028, Sigma-Aldrich). Samples were further incubated with secondary antibodies in 1% goat serum for 2 hours. Actin was stained using Alexa Fluor 555 Phalloidin or Alexa Fluor 488 Phalloidin (Thermo Fisher Scientific). The following antibodies were used: goat anti-mouse IgG Alexa Fluor 488 (11029, Thermo Fisher Scientific), goat anti-mouse IgG Alexa Fluor 594 (A11005, Thermo Fisher Scientific), goat anti-mouse IgG Alexa Fluor 647 (A21236, Thermo Fisher Scientific), goat anti-rabbit IgG Alexa Fluor 488 (A11034, Thermo Fisher Scientific), goat anti-rabbit IgG Alexa Fluor 546 (A11035, Thermo Fisher Scientific), and goat anti-rat IgG Alexa Fluor 546 (A11081, Thermo Fisher Scientific). Nuclei were counterstained using DAPI, and the coverslips were mounted using FluorSave reagent (Calbiochem). Images were acquired using the HC PL APO CS2 20×/0.75 DRY and HC PL APO CS 40×/0.85 DRY objectives of an SP8 confocal microscope (Leica). The morphology of mitochondria was quantified using the macro mitoMorph (*68*) for ImageJ. The intensity of fluorescent signal was quantified using the mean gray-value function of ImageJ. The length of axons in Boyden chambers was quantified using the AxonTracer plugin for ImageJ (*69*).

### RNA in situ hybridization

RNA in situ hybridization was performed following the instruction of the RNAscope 2.5 HD Fluorescent Reagent Kit (Advanced Cell Diagnostics). Neurons were fixed in 10% formalin, dehydrated and rehydrated in a gradient of 50-70-100% ethanol of 5 min, incubated with 1% Tween 20 for 15 min and with protease III for 10 min. Samples were then incubated with probes designed by the manufacturer to detect the corresponding mRNAs: *Atp5a1* (459311, Advanced Cell Diagnostics), *Pink1* (524081, Advanced Cell Diagnostics), and *Actb* (316741, Advanced Cell Diagnostics). Signal was amplified according to the instruction of the manufacturer, and samples were subjected to the immunofluorescence protocol described previously. Axons were straightened using the Straighten plugin of ImageJ, and the number of mRNA dots was automatically quantified using a macro developed by the CECAD imaging facility.

### FUNCAT assay

Newly synthetized proteins were labeled using the Click-iT HPG Alexa Fluor488 Protein Synthesis Assay Kit (Thermo Fisher Scientific). Neurons were incubated with 0.1 mM l-HPG (Thermo Fisher Scientific) for 30 min in l-methionine–free medium. Treatment with cycloheximide (0.1 mg/ml) for 3 hours was used as a negative control. Neurons were fixed in 4% FA for 15 min, permeabilized with 0.1% Triton X-100 for 10 min, incubated with Alexa Fluor azide 488 for 45 min, and then subjected to the immunofluorescence protocol previously described. Axons were selected, and the intensity of the fluorescence was measured using the multimeasure function of ImageJ.

### Apoptosis assay

Neurons were fixed in 4% FA for 15 min, permeabilized with 0.1% Triton X-100 for 10 min, and stained using the In Situ Cell Death Detection Kit, Fluorescein (Roche). Nuclei were counterstained using DAPI. Apoptosis was evaluated as the number of TUNEL^+^ cells respect to total DAPI^+^ nuclei.

### Live imaging

Live imaging experiments were performed using the LSM980 Airyscan 2 confocal microscope (Carl Zeiss Microscopy) equipped with the C-Apochromat 40×/1.20 W Korr or the Plan-Apochromat 63×/1.4 oil differential interference contrast objective.

#### Measurement of ATP

For imaging, samples were equilibrated in Hanks’ balanced salt solution containing B-27 supplement in presence of 25 mM glucose (for pcDNA-mitoAT1.03) or 25 mM galactose (pcDNA-AT1.03), and imaging was performed in the same medium. When indicated, samples were treated with oligomycin (0.01 mg/ml; Sigma-Aldrich)*.* Förster resonance energy transfer images were quantified using the fluorescent intensity function of ImageJ.

#### Mitochondrial potential

Neurons were grown in Boyden chambers as previously described. Membranes were incubated with 25 nM tetramethylrhodamine, methyl ester (Sigma-Aldrich) for 30 min. Samples were treated with oligomycin (0.01 mg/ml) and with 2 μM carbonyl cyanide *m*-chlorophenyl hydrazone (Sigma-Aldrich). Images from the axonal compartment were acquired 1 per min. The fluorescent signal from mitochondria was measured using the multimeasure function of ImageJ and normalized to the initial value.

#### Trafficking of mRNA

Primary motoneurons were co-transfected at DIV 4 with a 25:1 ratio of mRNA-MS2 and MCP-Halo. Twenty-four hours later, the cells were incubated with a final concentration of 16 nM Halo-Ligand JF 549 (Promega, GA1110) for 30 min, followed by one replacement of the medium. Neurites with visible mRNA dots were selected and a time lapse was taken with three *z*-slices per frame, one frame every 0.6 s and for 90 s overall. Movies were analyzed, on the one hand, by Kymographs that were generated from the maximum projected time lapses using ImageJ. In the Kymographs, mRNA tracks were manually traced, and the tracks were analyzed using the step-3 measure of the Kymolyzer macro (*70*). On the other hand, movies were analyzed with the automatic tracking module of the NIS-Elements AR software (Nikon). Only tracks with more than 20 frames were considered. A custom-made add-on MATLAB script was developed to analyze the type of movement and directionality of mRNA trajectories in neurites. In particular, to define the type of movement of each particle the MSD was calculated as follows: MSD(τ) ≤ [*x*(*t* + τ) − *x*(*t*)]2 + [*y*(*t* + τ) − *y*(*t*)] 2 > (Eq. 1), where *x* and *y* are the coordinates of the mRNA along the neurite, *t* and τ are the absolute and lag times, respectively, and the brackets represent the time average. This calculation was performed for τ = 25% of the total time of the trajectory (*71*). The MSD data were fitted with an anomalous diffusion model: MSD = *A*τα + *B* (Eq. 2), where *A* depends on the motion properties of the particle, *B* is the residual MSD, and the coefficient α is an indication of the particle motion type (*72*). Trajectories were classified as actively driven (α > 1.5), diffusive (0.9 < α < 1.1), or confined (α < 0.5). Last, to define the motion type based on the lateral displacement of mRNAs, the net displacement (ND) and lateral maximal displacement (LMD) were measured. ND is defined as the difference in *x* coordinates of the first point and the last point of the trace. LMD is defined as the difference between the first point and the most distal point during the trace. mRNAs with ND > 5 μm are defined directed; mRNAs with ND < 5 μm and LMD < 1 μm were defined stationary, while particles with ND < 5 μm and LMD > 1 μm were defined oscillatory (*73*).

### Real-time PCR

RNA from primary neurons was isolated using TRIzol (Thermo Fisher Scientific). cDNA was retrotranscribed from 1 μg of RNA using the SuperScript First-Strand Synthesis System (Life Technologies). Real-time polymerase chain reaction (PCR) was performed using the SYBR Green Master Mix (Applied Biosystems) with the QuantStudio 12K Flex Real-Time PCR System thermocycler (Applied Biosystems). The following primers were used: *ChAT* (forward) 5′-CCTGCCAGTCAACTCTAGCC-3′ and (reverse) 5′-TACAGAGAGGCTGCCCTGAG-3′; *Hb9* (forward) 5′-CCAAG-CGTTTTGAGGTGGC-3′ and (reverse) 5′-GGAACCAAATCTT-CACCTGAGTCT-3′; and *Gapdh* (forward) 5′-AGGTCGGTGTGA-ACGGATTTG-3′ and (reverse) 5′-TGTAGACCATGTAGTTGA-GGTCA-3′. The fold enrichment was calculated using the 2^(−ΔΔCt)^ formula. To quantify the mitochondrial DNA, the following primers were used: *mt-Co1* (forward) 5′-TGCTAGCCGCAGGCATTACT-3′ and (reverse) 5′-CGGGATCAAAGAAAGTTGTGTTT-3′; and *Rmrp* (forward) 5′-GCCTACACTGGAGTCGTGCTACT-3′ and (reverse) 5′-CTGACCACACGAGCTGGTAGAA-3′.

### Protein isolation and Western blot

Two million motoneurons were plated and grown for the indicated days. Proteins were extracted using radioimmunoprecipitation assay (RIPA) buffer (1% sodium cacodylate, 50 mM tris-HCl, 150 mM NaCl, 5 mM EDTA, and 1% Triton-X-100) and protease inhibitor cocktail (Sigma-Aldrich). For tissues, RIPA buffer was supplemented with 0.1% SDS. Proteins were quantified using standard Bradford (Bio-Rad) assay and subjected to SDS–polyacrylamide gel electrophoresis and transferred on polyvinylidene difluoride membranes. The following antibodies were used: monoclonal mouse anti–pan-actin (MAB1501R, Millipore) and polyclonal rabbit anti-CLUH (ARP70642_P050, Aviva).

### IP of CLUH in HeLa cells followed by MS

HeLa were lysed in IP buffer [50 mM tris-HCl (pH 7.4), 50 mM KCl, 0.1% Triton X-100, and freshly added protease inhibitor cocktail]. Bradford assay was used to quantify the protein concentration, and 400 μg of cell lysate was diluted in 250 μl of IP buffer and incubated with rabbit polyclonal anti-CLUH antibody (NB100-93306, Novus Biologicals) or rabbit IgG isotope control antibody (NB810-56910, Novus Biologicals) for 3 hours at 4°C with constant agitation. Afterward, the samples were incubated with 20 μl of prewashed Protein G Dynabeads (10003D, Thermo Fisher Scientific) with constant agitation at 4°C for 1 hour. This was followed by five washes with IP buffer on a magnetic stand, and the proteins were then heat eluted at 95°C for 5 min in 30 μl of SP3 lysis buffer (5% SDS in PBS). The supernatants were treated with 5 mM dithiothreitol at 55°C for 30 min and then with 40 mM chloroacetamide (CAA) at room temperature for another 30 min in the dark. After centrifugation, supernatants were processed by the CECAD Proteomics Facility. All samples were analyzed on a Q-Exactive Plus (Thermo Fisher Scientific) mass spectrometer that was coupled to an EASY nLC 1000 or 1200 UPLC (Thermo Fisher Scientific), as previously described (*19*). Student’s *t* tests were calculated in Perseus (version 1.6.15.0) after removal of decoys and potential contaminants. Data were filtered for at least four of the four values in at least one condition. Remaining missing values were imputed with random values from the lower end of the intensity distribution using Perseus defaults. Proteins were considered significant interactors with *P* < 0.05, *q* < 0.05, and fold change > 0.40. Kyoto Encyclopedia of Genes and Genomes pathway analysis of significantly enriched proteins was performed using the EnrichR webtool (*59*).

### Proteomics of primary neurons cultured in Boyden chambers

Neurons were grown in Boyden chambers for 6 days. Axons were scraped, and neurons were lysed with 4% SDS in 100 mM Hepes (pH 8.5) and snap-frozen into liquid nitrogen. Samples were thawed, and proteins were reduced [10 mM tris(2-carboxyethyl)phosphine] and alkylated (20 mM CAA) in the dark for 45 min at 45°C. Then, samples were heated to 10 min of incubation at 70°C on a ThermoMixer (shaking, 550 rpm). For neuron samples, the protein concentration was determined using the 660-nm Protein Assay (Thermo Fisher Scientific, no. 22660), and 20 μg of protein was subjected to tryptic digestion. For axon samples, the total volume was used. Samples were subjected to an SP3-based digestion (*74*). Washed SP3 beads {SP3 beads [Sera-Mag magnetic carboxylate modified particles (hydrophobic, GE44152105050250) and Sera-Mag magnetic carboxylate modified particles (hydrophilic, GE24152105050250)] from Sigma-Aldrich} were mixed equally, and 3 μl (soma) and 1 μl (axon) of bead slurry were added to each sample. Acetonitrile was added to a final concentration of 50% and washed twice using 70% ethanol (*V* = 200 μl) on an in-house made magnet. After an additional acetonitrile wash (*V* = 200 μl), 5 μl (soma) or 0.5 μl (axon) of digestion solution [10 mM Hepes (pH 8.5) containing 0.5 μg of trypsin (Sigma-Aldrich) and 0.5 μg of LysC (Wako) per microliter] was added to each sample and incubated ON at 37°C. Peptides were desalted on a magnet using 2× 200 μl of acetonitrile. Peptides were eluted in 10 μl of 5% DMSO in liquid chromatography–MS (LC-MS) water (Sigma-Aldrich) in an ultrasonic bath for 10 min. Formic acid and acetonitrile were added to a final concentration of 2.5 and 2%, respectively. The protocol was automated using CyBio Felix (Analytik Jena) and Mosquito LV (SPT Labtech) liquid handler. Samples were stored at −20°C before subjection to LC–tandem MS (MS/MS) analysis.

#### LC and MS

LC-MS/MS instrumentation consisted of an Easy-LC 1200 (Thermo Fisher Scientific) coupled via a nano-electrospray ionization source to an Exploris 480 mass spectrometer (Thermo Fisher Scientific, Bremen, Germany). An in-house packed column (inner diameter, 75 μm; length, 20 cm) was used for peptide separation. A binary buffer system (A, 0.1% formic acid; and B, 0.1% formic acid in 80% acetonitrile) was applied as follows: linear increase of buffer B from 4 to 27% within 40 min, followed by a linear increase to 45% within 5 min. The buffer B content was further ramped to 65% within 5 min and then to 95% within 5 min. Buffer B (95%) was kept for a further 5 min to wash the column. Before each sample, the column was washed using 5 μl of buffer A, and the sample was loaded using 8 μl of buffer A.

The radio frequency lens amplitude was set to 55%, the capillary temperature was 275°C, and the polarity was set to positive. MS1 profile spectra were acquired using a resolution of 60,000 [at 200 mass/charge ratio (*m*/*z*)] at a mass range of 320 to 1150 *m*/*z* and an automatic gain control target of 1 × 10^6^.

For MS/MS-independent spectra acquisition, 48 equally spaced windows were acquired at an isolation *m*/*z* range of 15 thomson, and the isolation windows overlapped by 1 Th. The fixed first mass was 200 *m*/*z*. The isolation center range covered a mass range of 357 to 1060 *m*/*z*. Fragmentation spectra were acquired at a resolution of 15,000 at 200 *m*/*z* using a maximal injection time of 22 ms and stepped normalized collision energies of 26, 28, and 30. The default charge state was set to 3. The AGC target was set to 3e6 (900%, Exploris 480). MS2 spectra were acquired in centroid mode. Field Asymmetric Ion Mobility Spectrometry was enabled and used at a compensation voltage of −50 for all samples using an inner electrode temperature of 89°C and an outer electrode temperature of 99.5°C.

#### Data analysis

DIA-NN (Data-Independent Acquisition by Neural Networks) version 1.8 (*75*) was used to analyze data-independent raw files. Axon and soma samples were analyzed separately. The spectral library was created using the reviewed-only UniProt reference protein (*M. musculus*, 17,029 entries) with the “Deep learning-based spectra and retention time prediction” turned on. Protease was set to trypsin, and a maximum of one miss cleavage was allowed. N-terminal M excision was set as a variable modification, and carbamidomethylation at cysteine residues was set as a fixed modification. The peptide length was set to 7 to 30 amino acids, and the precursor *m*/*z* range was defined from 340 to 1200 *m*/*z*. The option “Quantitative matrices” was enabled. The false discovery rate was set to 1% and the mass accuracy (MS2 and MS1), as well as the scan window was set to 0 (automatic inference via DIA-NN). Match between runs was enabled. The neuronal network classifier worked in “double pass mode,” and protein interference was set to “Isoform IDs.” The quantification strategy was set to “robust LC (high accuracy),” and cross-run normalization was defined as “RT-dependent.” The “pg” (protein group) output [MaxLFQ intensities; (*76*)] was further processed using Instant Clue (*77*) including a pairwise one-sample *t* test using the isolation and litter-batch–derived fold change between NKO and WT. MitoCarta 3.0 and UniProt-based gene ontology annotations were added and used for filtering. Volcano plots were generated using Prism 8.3.0 (GraphPad Software LLC). Gene ontology analysis of significantly enriched proteins (*P* < 0.05; fold change as indicated in the Results) was performed using the EnrichR webtool (*59*).

### Software and statistical analysis

Templates for images or schemes were generated using Biorender. Prism 8.3.0 (GraphPad Software LLC) was used for statistical analysis. In graphs, average and SD (± SD) are reported, where each dot represents one independent experiment blind to the experimenters. The numbers of experiments and samples per experiment are reported in the figure legends. Normality was assessed using the Shapiro-Wilk test. *P* values and statistical tests are reported in the figures and the figure legends.
