# Supplementary material for: CLUH maintains functional mitochondria and translation in motoneuronal axons and prevents peripheral neuropathy
Source: Sci Adv. 2024 May 29;10(22):eadn2050. doi: 10.1126/sciadv.adn2050 (PMC11135423; doi:10.1126/sciadv.adn2050)
Supplement: Supplementary file 1 — Figs. S1 to S9 Legends for movies S1 to S4 Legends for tables S1 to S3 [file sciadv.adn2050_sm.pdf]

Science Advances
AAAS

Supplementary Materials for

CLUH maintains functional mitochondria and translation in motoneuronal axons and prevents peripheral neuropathy

Marta Zaninello *et al.*

Corresponding author: Elena I. Rugarli, elena.rugarli@uni-koeln.de

Sci. Adv. **10**, eadn2050 (2024)
DOI: 10.1126/sciadv.adn2050

The PDF file includes:

Figs. S1 to S9
Legends for movies S1 to S4
Legends for tables S1 to S3

Other Supplementary Material for this manuscript includes the following:

Movies S1 to S4
Tables S1 to S3

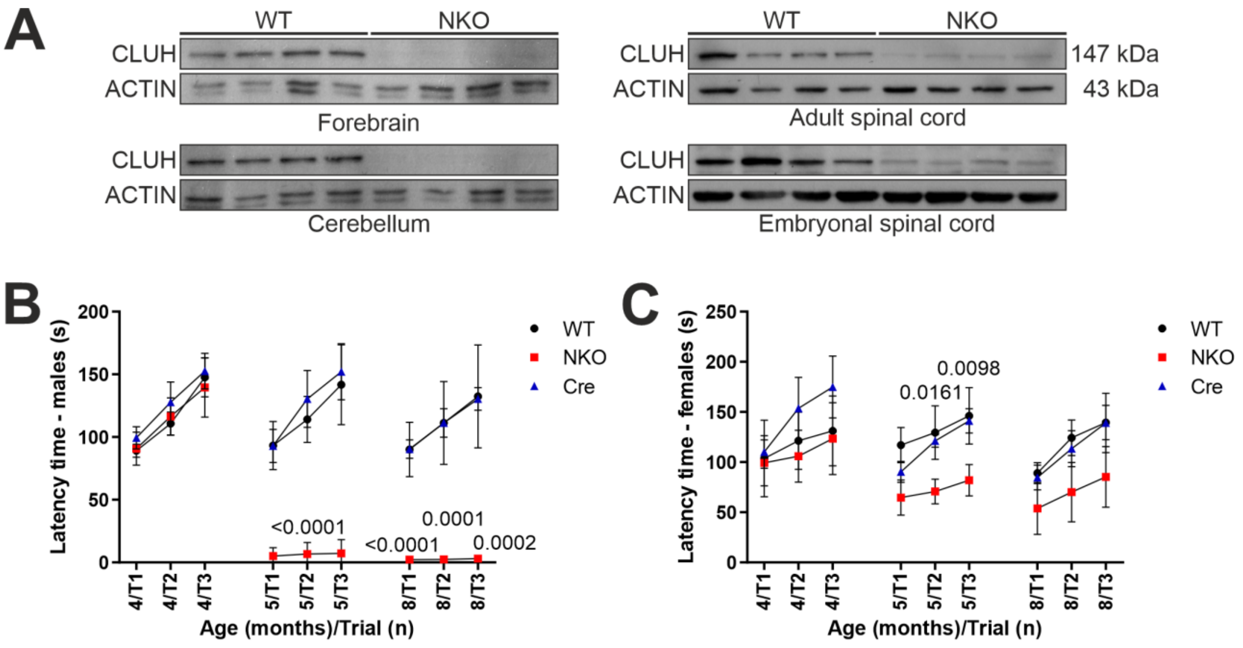

Fig. S1. NKO mice display an impaired rotarod performance

(**A**) Western blots depicting CLUH levels in forebrain, cerebellum and spinal cord of WT and NKO mice aged 5 months and spinal cord of WT and NKO E13.5 embryos. (**B**, **C**) Latency time to fall from a rotarod apparatus for male (B) and female (C) mice of the indicated genotypes. Data represent mean ± SD of 6-11 mice per group. Statistical significance was determined by one-way ANOVA followed by Dunn's and Dunnett's multiple comparison tests.

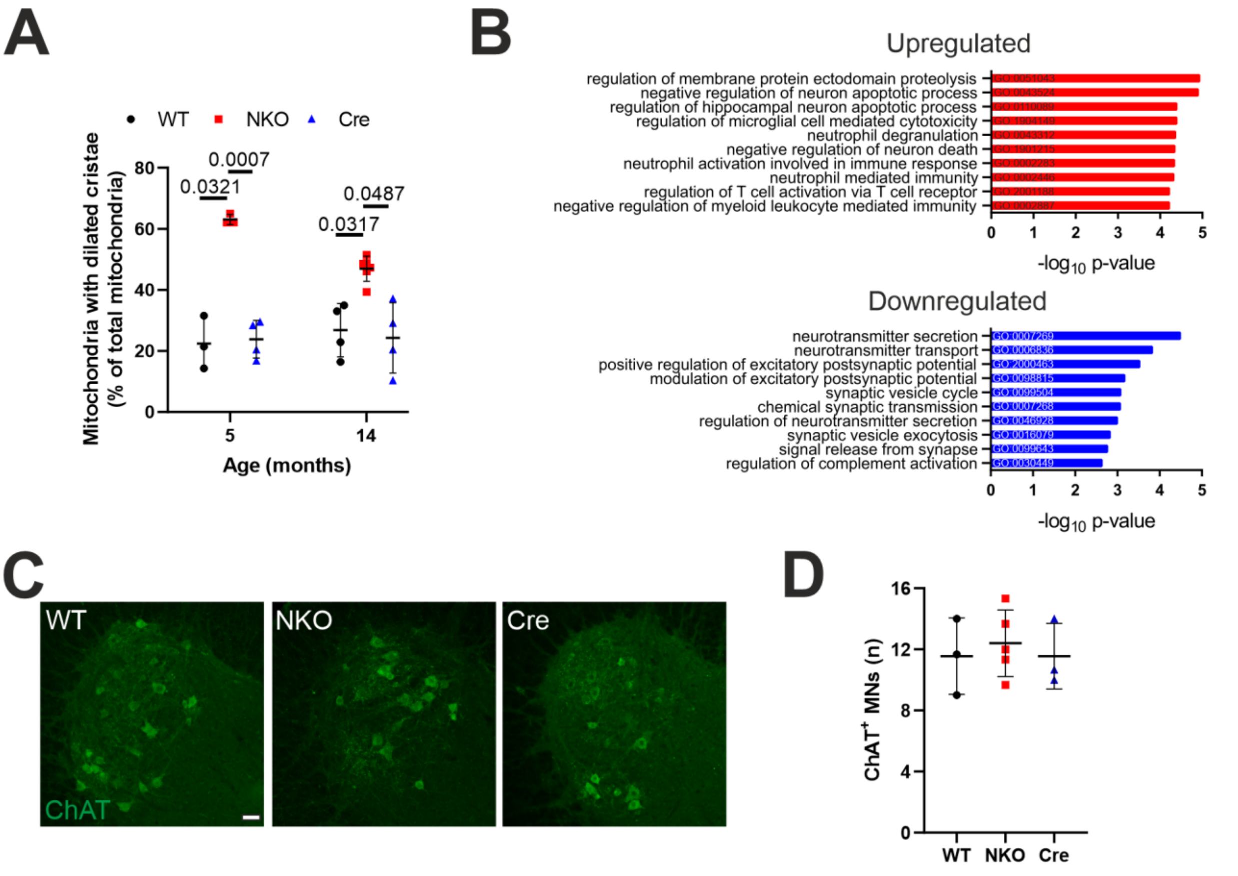

Fig. S2. NKO motoneurons do not die *in vivo*, but show signs of axonopathy

(**A**) Quantification of the percentage of mitochondria with dilated cristae in axons of the tibial branch of the sciatic nerve of mice at 5 and 14 months of age. Data represent mean ± SD of 3-6 mice (55-219 mitochondria per mouse). Statistical significance was determined by one-way ANOVA followed by Dunnett's multiple comparison test. (**B**) Gene ontology biological process (GOBP) analysis of upregulated (up, red) and downregulated (bottom, blue) transcriptome changes in the sciatic nerves of 5 months old male NKO mice compared to WT mice. Analysis was done using the EnrichR webtool. (**C**) Anterior spinal cord stained for Choline Acetyltransferase (ChAT, green) in mice of indicated genotypes at 14 months of age. Bar, 50 µm. (**D**) Quantification of the number of motoneurons in experiments shown in (C). Data represent mean ± SD of 3-5 mice (4-6 sections per mouse).

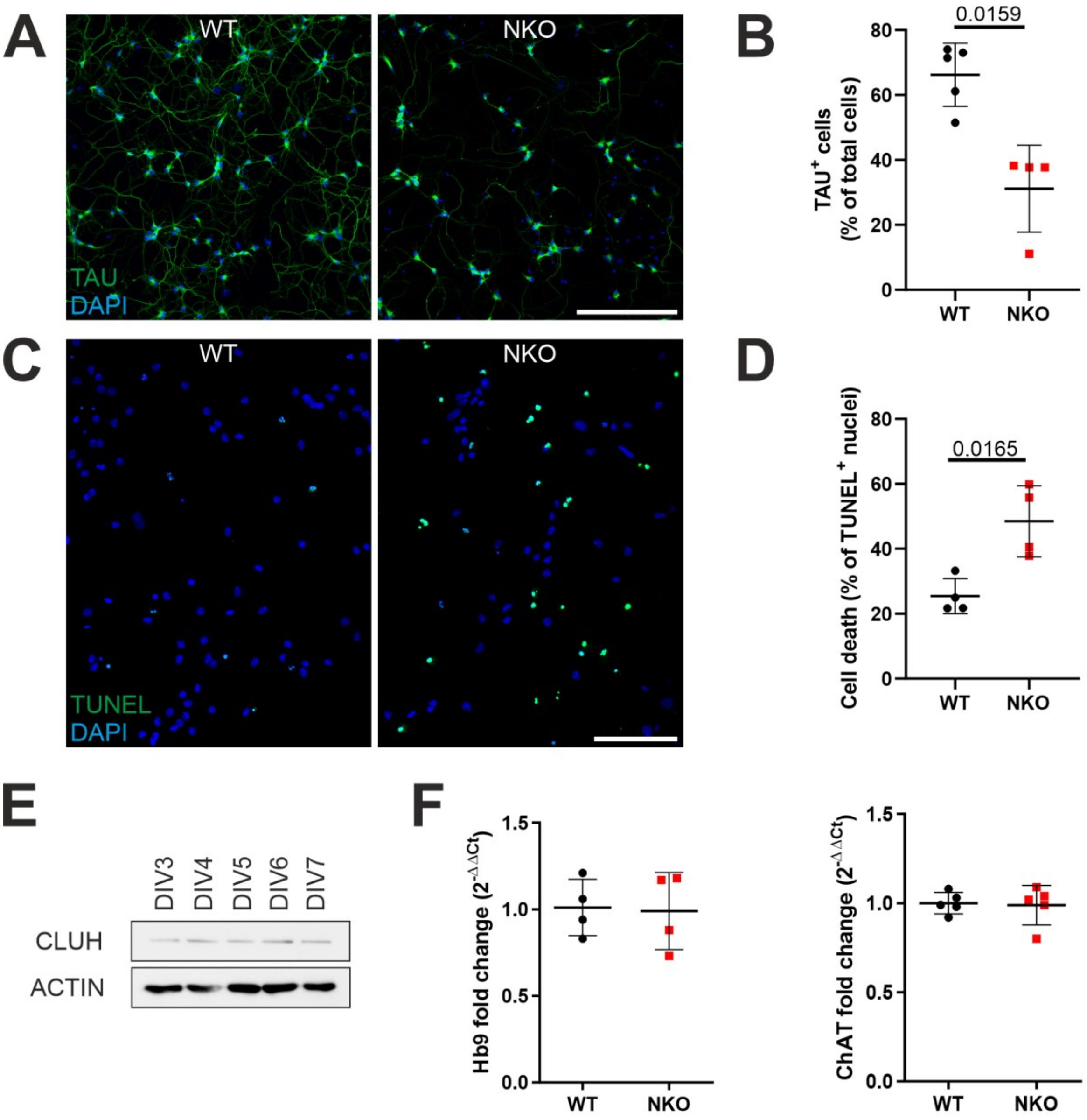

Fig. S3. Phenotypes of NKO motoneurons

(**A**) Primary spinal motoneurons at DIV 10 stained with TAU (green) and DAPI (blue). Bar, 50 µm. (**B**) Quantification of the number of TAU^+ neurons in experiments as in (A). Data represent mean ± SD of 4-5 mice (20 fields per mouse). Statistical significance was determined by Mann-Whitney test. (**C**) Nuclei (DAPI, blue) and apoptotic nuclei stained using TUNEL assay (green) of primary motoneurons at DIV 10. Bar, 50 µm. (**D**) Quantification of apoptotic nuclei in experiments as in (C). Data represent mean ± SD of 4 mice (10 fields per mouse). Statistical significance was determined by Welch's t test. (**E**) Western blot depicting CLUH levels in

primary motoneurons from DIV 3 to DIV 7. (**F**) Quantification of the expression of Hb9 and ChAT in primary motoneurons at DIV 5. Data represent mean ± SD of 4-5 mice.

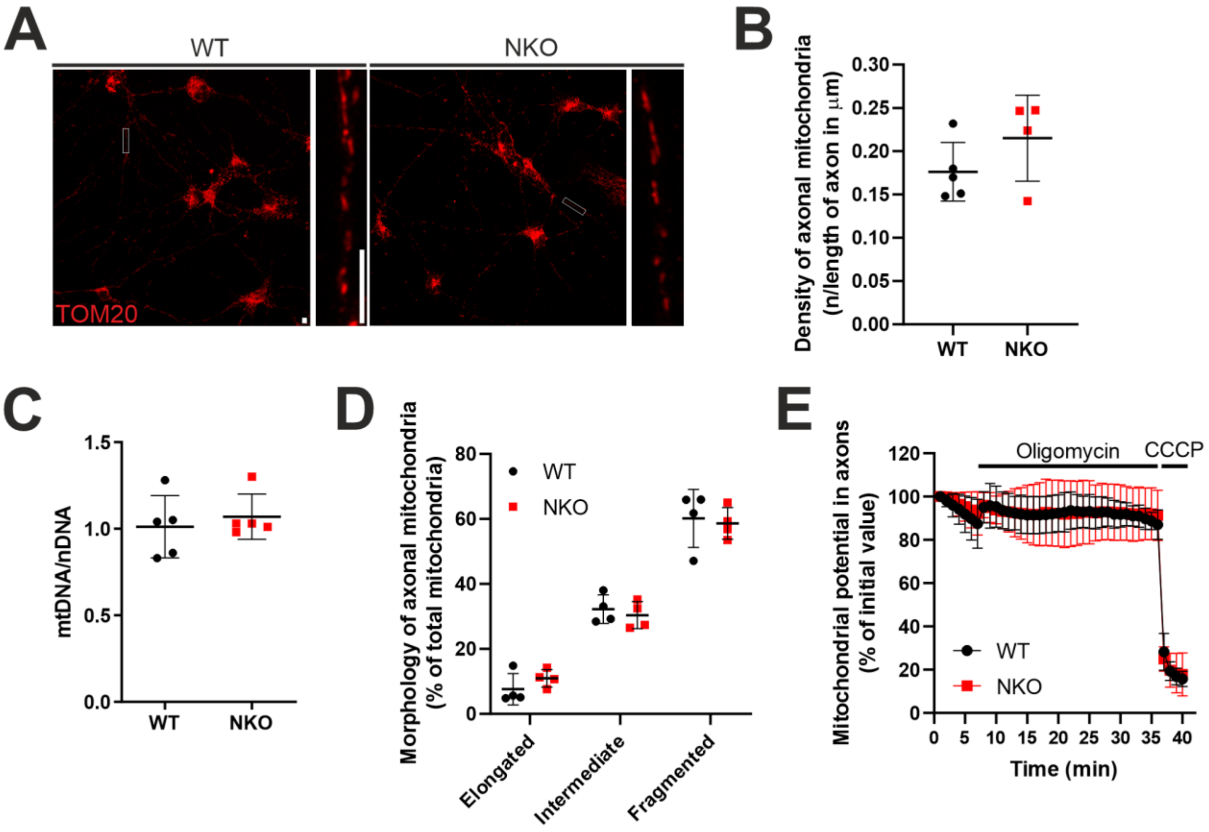

Fig. S4. NKO mitochondria are comparable to WT mitochondria in respect to clustering, morphology and membrane potential

(**A**) Primary motoneurons stained with TOM20 (red). Representative axons are straightened and magnified on the right of each panel. Bars, 5 µm. (**B**) Quantification of the density of mitochondria in axons in experiments shown in (A). Data represent mean ± SD of 4-5 cultures (24-35 axons per culture). (**C**) Quantification of the abundance of mitochondrial DNA (mtDNA) to nuclear DNA (nDNA). Data represent mean ± SD of 5 motoneuronal cultures. (**D**) Quantification of the morphology of mitochondria in axons in experiments shown in (A). Data represent mean ± SD of 4 cultures (28-30 axons per culture). (**E**) Quantification of the mitochondrial membrane potential of axons of primary motoneurons loaded with TMRM. Neurons were treated with oligomycin after 5 minutes and CCCP after 35 minutes. Data represent mean ± SD of 4 WT and 5 NKO cultures.

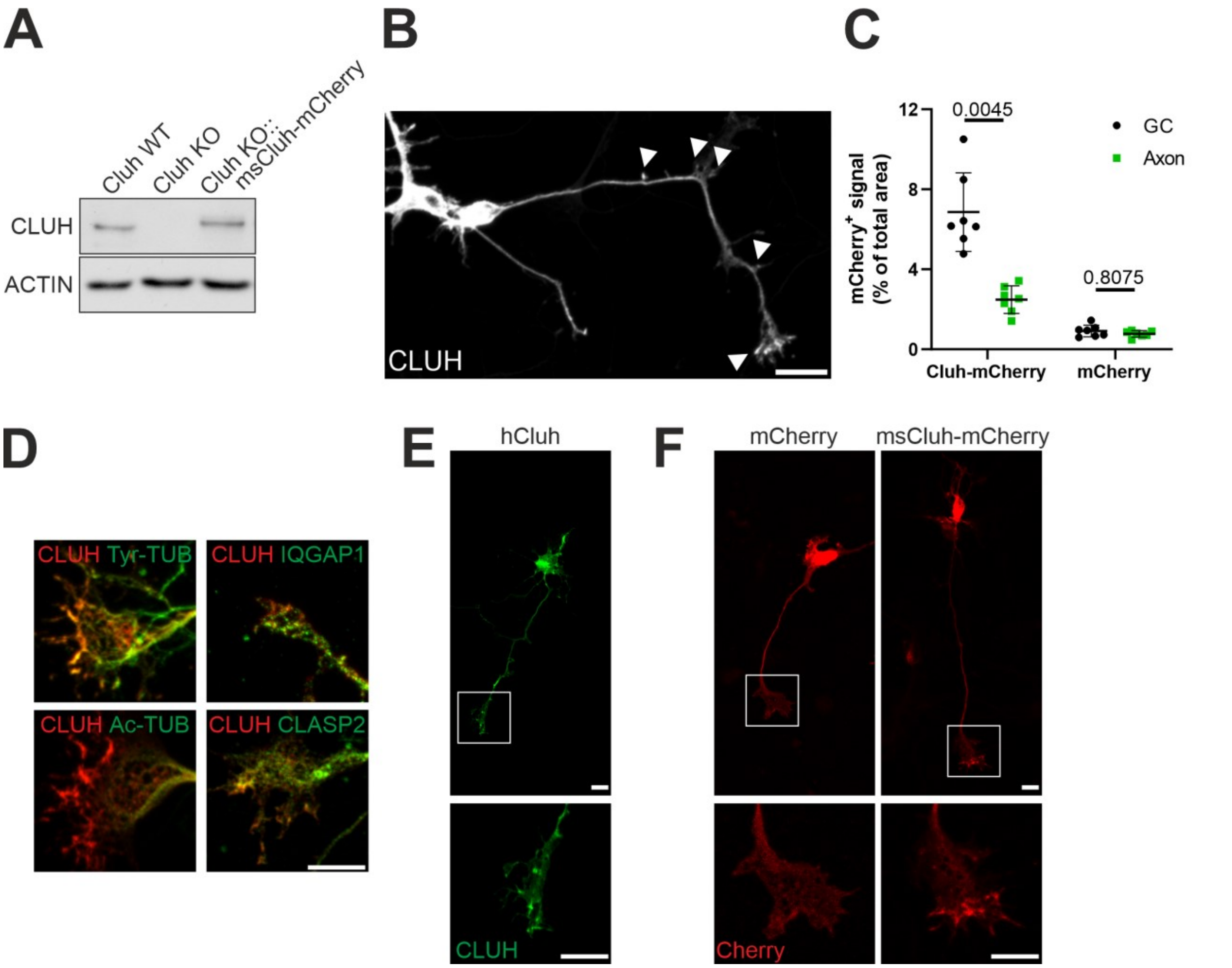

Fig. S5. Overexpressed Cluh-mCherry localizes to somas, axons and GCs

(**A**) Western blot depicting CLUH levels in WT or KO mouse embryonic fibroblasts transfected with ms*Cluh*-mCherry (ms, mouse). (**B**) Overexpression of CLUH-mCherry in a primary spinal motoneuron shows signal in the soma, in protrusions and the GCs (arrowheads). Bar, 20 µm. (**C**) Area of CLUH$^+$ signal in GCs in respect to distal axons from images of the experiment shown in Fig. 4G. Data represent the mean ± SD of 7 WT independent cultures (6-12 cells per culture). Statistical significance was determined by one-way ANOVA followed by Dunnett's multiple comparison test. (**D**) GCs of primary motoneurons transfected with ms*Cluh*-mCherry (red) and stained for proteins of the cytoskeleton (green). Ac-TUB, acetylated tubulin, Tyr-TUB, tyrosinated tubulin. Bar, 10 µm. (**E**) A primary motoneuron transfected with untagged human *CLUH* and stained with an anti-human CLUH antibody (green). The boxed area containing a GC

is enlarged in the lower image. Bars, 10 µm. (**F**) Primary motoneurons transfected with mCherry or ms*Cluh*-mCherry. Boxed areas showing GCs are enlarged in the lower image. Bars, 10 µm.

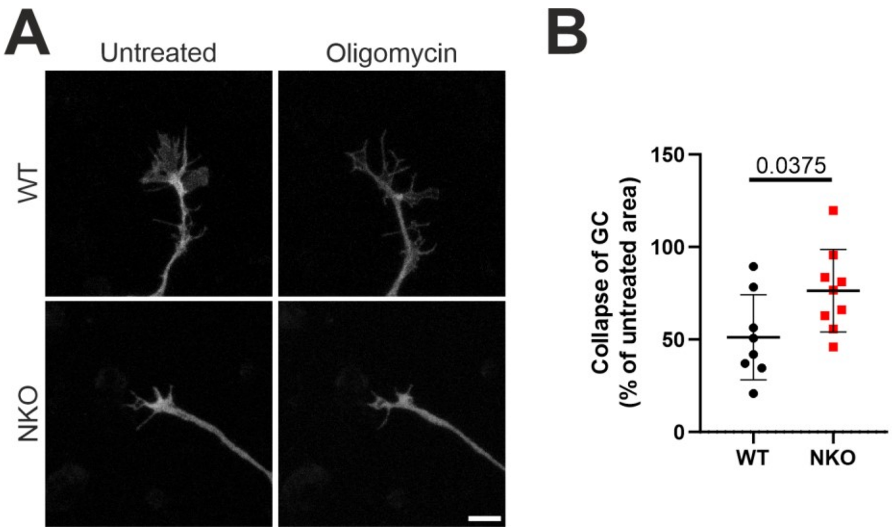

Fig. S6. Inhibition of ATP synthase induces the collapse of WT GCs but not NKO GCs
(**A**) GCs transfected with GFP (grey) were imaged before and after addition of oligomycin. Bar, 10 µm. (**B**) Quantification of the percentage of reduction of the area of GCs after oligomycin treatment (15 min after administration) in experiments as in (A). Data represent mean ± SD of 8-9 GCs from different mice. Statistical significance was determined by Welch's t test.

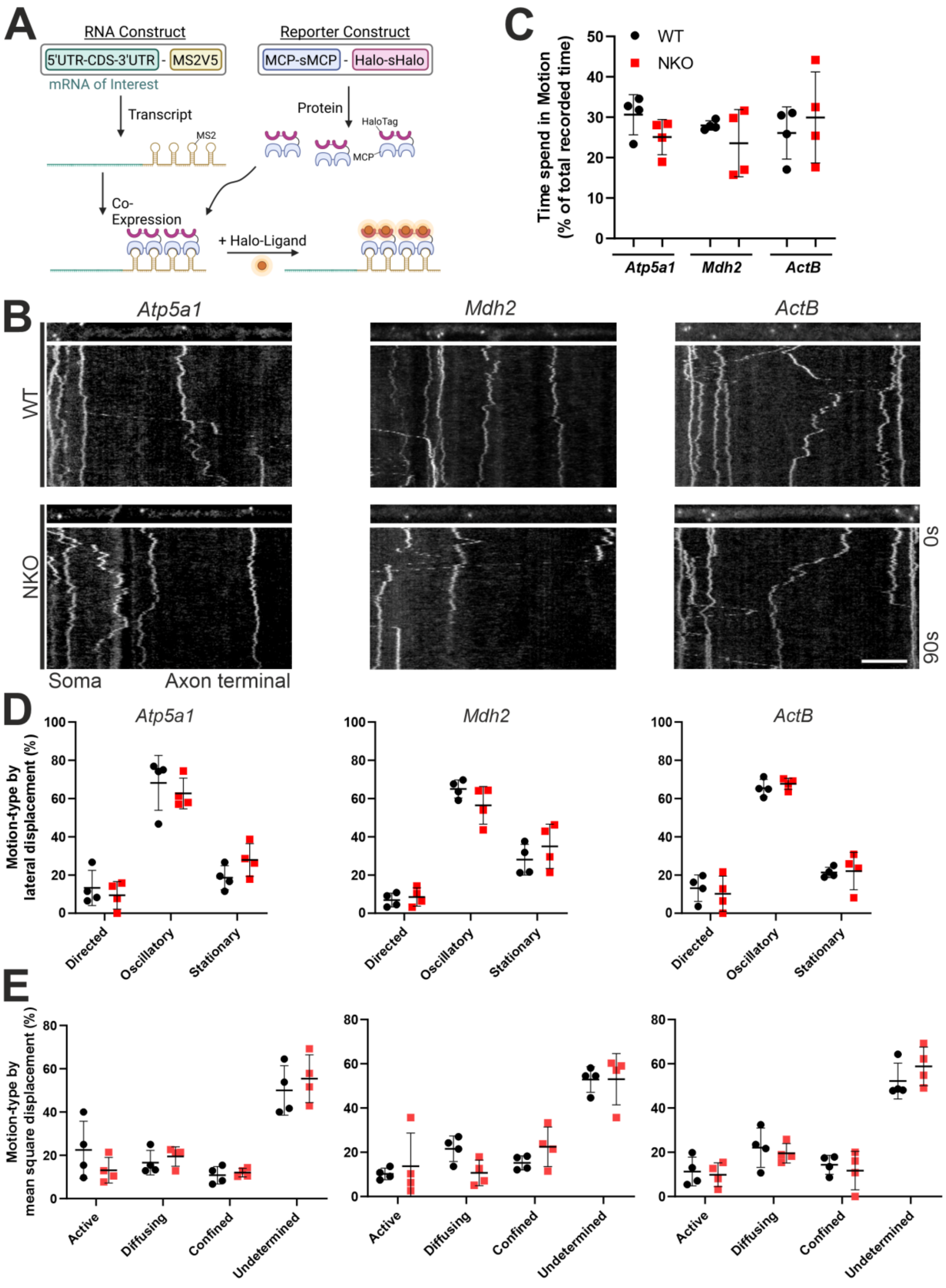

Fig. S7. CLUH does not alter the transport of CLUH target mRNAs

(**A**) The MS2-MCP system. An “RNA construct” with the target mRNA and a 3’ attached MS2V5 sequence (RNA-MS2) is co-expressed with a “reporter construct”, coding for the MCP coating protein tagged with HaloTag as the reporter protein (MCP-Halo). After the RNA construct is transcribed, MS2 stem loops form, allowing MCP-Halo to bind. Addition of Halo-ligand enables the visualization of the mRNA. Figure created with Biorender.com. (**B**) Kymograph of *Atp5a1*, *Mdh2* and *ActB* mRNAs in axons of primary motoneurons using the MS2-MCP system depicted in (A). The first frame of the recording is shown as a straightened segment in the image above the kymograph. Bar, 5 µm. (**C**) Time spent in motion relative to the total tracked time and using a speed cut-off of more than 0.1 µm/s in experiments as in (B). (**D**) Division in three different motion-types (directed, oscillatory, stationary) as defined by total and maximal lateral displacement over the whole track or (**E**) by the mean square displacement in the first 25% of the track (active, diffusing, confined) in experiments as in (B). Data represents the mean ± SD of 4 cultures (10-93 mRNA dots per culture).

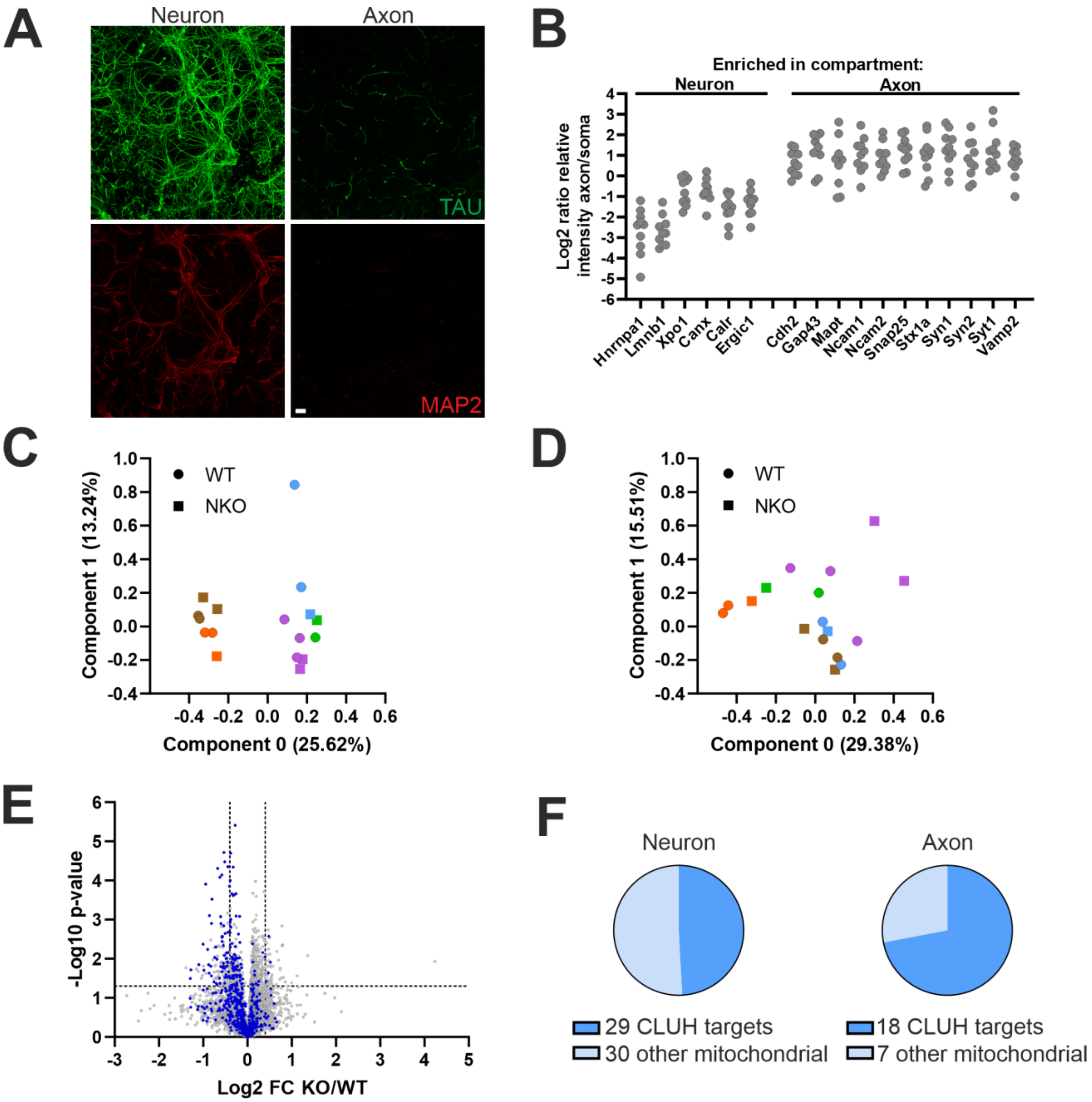

Fig. S8. Validation of the proteomics from Boyden chambers

(**A**) Neuron (left panel) and axon (right panel) compartments of Boyden chambers. Primary motoneurons are stained with MAP2 (red) and TAU (green). Bar, 25 µm. (**B**) Ratio of the protein intensities of the neuron and the axon compartments per sample. Hnrnpa1, Lmnb1 and Xpo1 were considered as proteins enriched in nuclei, Canx and Calr in ER and Ergic1 in Golgi. (**C**, **D**) Principal component analysis of proteomics in the neuron (C) and axon (D) compartment. Each day of collection of samples is depicted by a different colour. (**E**) Volcano plot showing protein changes in NKO versus WT neuron compartments of Boyden chambers. Mitochondrial proteins

are highlighted in blue. (**F**) Pie charts depicting all measured downregulated mitochondrial proteins and the percentage of putative CLUH targets in the neuron (left) and axon (right) compartments.

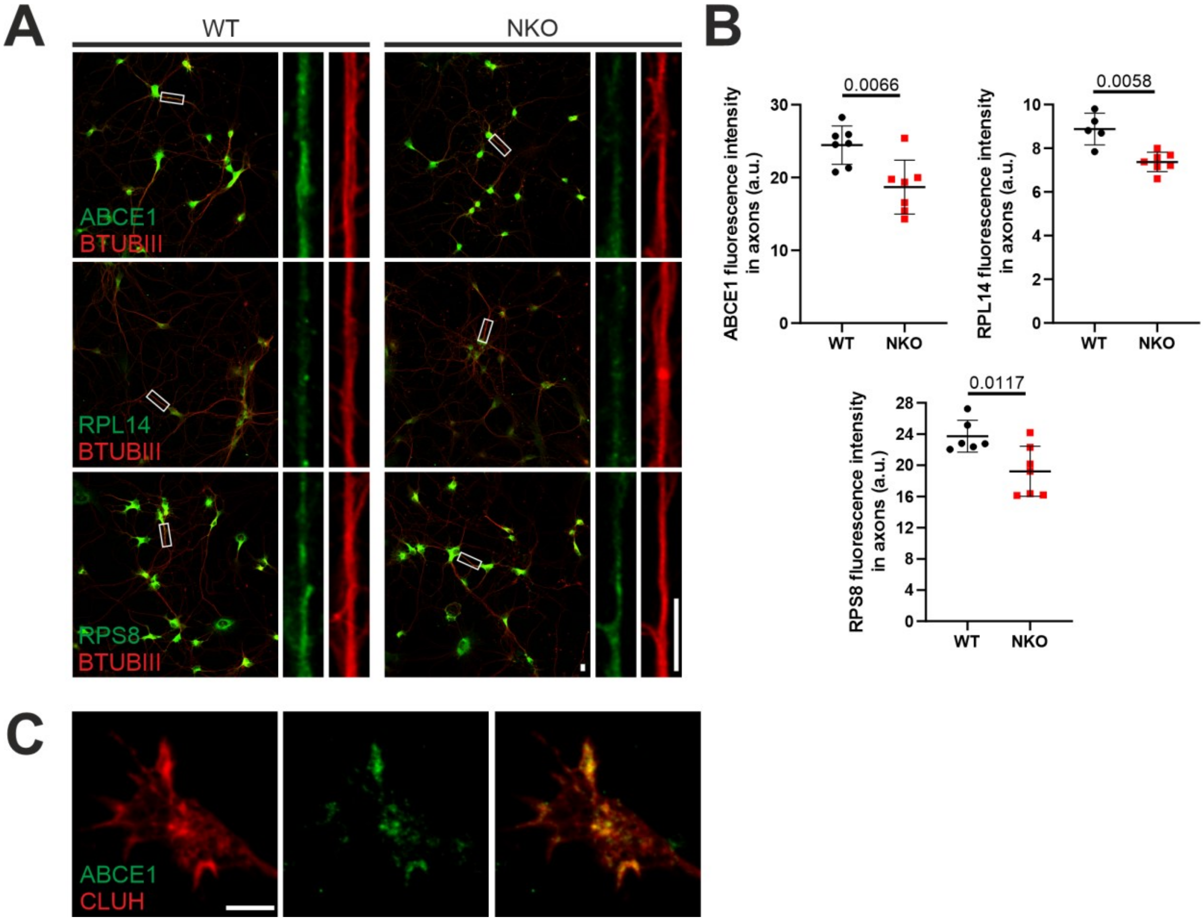

Fig. S9. ABCE1, RPL14 and RPS8 are reduced in NKO axons

(**A**) Primary spinal motoneurons stained for ABCE1, RPL14 or RPS8 (green) and β-TUBULIN III (red). Axons are straightened and magnified next to the field showing motoneurons. Bars, 10 µm. (**B**) Quantification of the intensity of the fluorescence of ABCE1 (upper left graph), RPL14 (upper right graph) and RPS8 (lower graph) in axons of experiments as in (A). Data represent the mean ± SD of 5-7 cultures (30-58 axons per culture). Statistical significance was determined by Welch's t test. (**C**) Single confocal plane of a GC transfected with ms*Cluh*-mCherry (red) and stained for ABCE1 (green).

Movie S1.
Recordings of WT and NKO male mice aged 4 months performing the walking beam test.

Movie S2.
Recordings of WT and NKO male mice aged 5 months performing the walking beam test.

Movie S3.
Recordings of WT and NKO male mice aged 8 months performing the walking beam test.

Movie S4.
Live imaging of WT and NKO GCs treated with oligomycin. Relative to Fig. S6.

Table S1. (separate file)
RNAseq results of sciatic nerves from WT and NKO male mice aged 5 months.

Table S2. (separate file)
MS results of WT and NKO neurons and axons separated using Boyden chambers.

Table S3. (separate file)
MS results of CLUH immunoprecipitation of in HeLa cells.
